# Small extracellular vesicles from DENV2-infected C6/36 cells show viral infection *in vitro* and *in vivo*

**DOI:** 10.1080/22221751.2025.2608403

**Published:** 2026-01-27

**Authors:** Carlos D. Cordero-Rivera, Magda L. Benítez-Vega, Selvin N. Palacios-Rápalo, José De Jesús Bravo-Silva, Ricardo Jiménez-Camacho, Jonathan Hernández-Castillo, Marcos Pérez-García, Carlos N. Farfan-Morales, Luis A. De Jesús-González, José M. Reyes-Ruiz, Juan F. Osuna-Ramos, Fernando Medina-Ramirez, Daniel Talamás-Lara, Raymundo Cruz-Pérez, Arturo Reyes-Sandoval, Rosa M. Del Angel

**Affiliations:** a Departamento de Infectómica y Patogénesis Molecular, Centro de Investigación y de Estudios Avanzados del Instituto Politécnico Nacional (CINVESTAV-IPN), Ciudad de México, México; b Unité de Virologie Structurale, Institut Pasteur, Paris, France; c Departamento de Ciencias Naturales, Universidad Autónoma Metropolitana (UAM), Unidad Cuajimalpa, Ciudad de México, México; d Laboratorio de Virología Molecular, Unidad de Investigación Biomédica de Zacatecas, Instituto Mexicano del Seguro Social (IMSS), Zacatecas, México; e División de Investigación en Salud, Unidad Médica de Alta Especialidad, Hospital de Especialidades No. 14, Centro Médico Nacional “Adolfo Ruiz Cortines”, Instituto Mexicano del Seguro Social (IMSS), Veracruz, México; f Facultad de Medicina de la Universidad Autónoma de Sinaloa (UAS), Culiacán, México; g Laboratorios Centrales, Centro de Investigación y de Estudios Avanzados del Instituto Politécnico Nacional (CINVESTAV-IPN), Ciudad de México, México; h Dirección General, Instituto Politécnico Nacional (IPN), Ciudad de México, México

**Keywords:** Small extracellular vesicles (sEVs), dengue virus (DENV), viral transmission, murine models (AG129 and CD-1 mice), dengue pathogenesis

## Abstract

Dengue, transmitted by *Aedes* mosquitoes, can progress to severe symptoms like hemorrhagic fever and shock syndrome. While the virus and host immune response contribute to severity, other factors, such as small extracellular vesicles (sEVs), may play a role. sEVs mediate intercellular communication by transferring cellular components; however, their role *in vivo* infection remains unclear. We isolated and characterized sEVs from DENV-infected C6/36 mosquito cells, finding that they interact with mammalian cells and internalize the content. Using sEVs populations (with a size between 100 and 200 nm), we demonstrated enhanced infection in *in vitro* and *in vivo* murine models, including immunocompetent and immunosuppressed mice, which developed severe dengue-like symptoms. Our study reveals that sEVs from DENV-infected mosquito cells contribute to dengue pathogenesis, inducing severe symptoms in *in vivo* models, highlighting their potential role in disease progression and severe outcomes.

## Introduction

Dengue is one of the most common and dangerous viral diseases caused by one of the four serotypes of the *Orthoflavivirus denguei* (DENV) *.* DENV is transmitted to humans through the bites of *Aedes* mosquitoes, including *Aedes albopictus* and *Aedes aegypti*. According to the World Health Organization (WHO), dengue infection is now endemic in 176 countries, affecting approximately 3.6 billion people (40% of the world's population). In 2024, the highest number of reported dengue cases was recorded, with an estimated 14 million cases and over 10,000 dengue-related deaths worldwide.

Patients with dengue infection typically progress through three typical clinical phases: the febrile phase, the critical phase, and the recovery phase. The disease begins with an incubation period of 3–7 days, followed by a sudden onset of high fever and marked viremia, characteristic of the febrile phase. Some patients progress to the critical phase, which lasts 24–48 h (hours) and is associated with plasma leakage and increased hematocrit. However, in extreme cases, the infection can trigger severe clinical manifestations known as severe dengue, characterized by transient increases in vascular permeability due to endothelial dysfunction, leading to plasma leakage, hypovolemic shock, and hemorrhage. A marked disruption of blood capillaries in vital organs (including the liver, spleen, intestines, heart, and central nervous system (CNS)) has been observed. Vascular hyperpermeability and plasma leakage are hallmarks of severe dengue [1], and several studies have sought to elucidate the host – or virus-related factors contributing to its development [2,3]. However, despite extensive research, no single host – or virus-related factor has been definitively identified as the sole cause of severe dengue. In this context, extracellular vesicles (EVs) have emerged as promising candidates [4].

EVs are nanosized non-replicative membrane-bound particles secreted by cells and carrying diverse bioactive molecules (including proteins, lipids, and nucleic acids) that modulate recipient cell behavior or induce phenotypic changes [5]. The International Society for Extracellular Vesicles (ISEV) has proposed Minimal Information for Studies of EV (MISEV), which delineates the characterization of EVs based on their physical properties, such as size, cell origin, and molecular composition [6]. Small EVs (sEVs), commonly referred to as exosomes, are typically less than 200 nm in diameter, whereas ectosomes (also known as microparticles or microvesicles) comprise Large EVs (lEVs) with diameters exceeding 200 nm. Apoptotic bodies (ApoBDs), the largest category, exceed 1,000 nm in diameter. In terms of biogenesis, sEVs originate from the endosomal pathway through the exocytosis of intraluminal vesicles (ILVs) from multivesicular bodies (MVBs), whereas lEVs bud directly from the plasma membrane, and ApoBDs are released from apoptotic cells. Molecularly, sEVs are enriched in proteins involved in vesicle formation and trafficking, such as tetraspanins (CD9, CD63, and CD81), while lEVs contain plasma membrane proteins like annexin V, and APoBDs are characterized by the presence of caspases, p53, and cytochrome C [7].

EVs play a crucial role in intercellular communication and have been implicated in various physiological and pathological processes, including viral infection. Viruses can hijack EVs to promote dissemination, evade immune response, and contribute to pathogenesis. Infected cells release EVs that contain viral components, which modulate the host immune system. In the context of DENV and sEVs, recent studies have revealed that DENV-infected mosquito cells enhance the formation and release of sEVs containing viral genetic material and proteins, which can infect both mosquito and mammalian cells [8]. Our research group has determined that these sEVs can act as Trojan horses, facilitating viral dissemination [9]. The sEVs from DENV-infected mosquito cells exhibit an altered protein cargo [10], enhancing infection by transferring viral components to naïve cells [8]. Moreover, mosquito salivary glands secrete sEVs [10] loaded with subgenomic flaviviral RNA (sfRNA), which increases viral infectivity by inhibiting type I and III interferon signaling [11]. However, their role in *in vivo* infection remains unexplored. Therefore, the objective of the work was to isolate sEVs from mosquito cells and to determine their role in *in vivo* infections.

## Materials and methods details

### Experimental model and study participant details

#### Cell lines

*Aedes albopictus* larvae clone C6/36 HT (C6/36) cells were cultured in Minimal Essential Medium (MEM) supplemented with 10% EVs-depleted FBS, vitamins, 0.034% sodium bicarbonate, 100 μg/mL streptomycin, and 100 U/mL penicillin. Cells were maintained at 32°C and in a humidified atmosphere without CO_2_. The human hepatocellular carcinoma cell line (Huh-7) was grown in advance Dulbecco´s Modified Eagle Medium (DMEM) (complete medium) supplemented with 2 mM glutamine, 7% fetal bovine serum (FBS), high glucose (4 g/L), Amphotericin B (1000X), penicillin (50 U/mL) and streptomycin (50 mg/mL) at 37°C and a 5% CO_2_ humidified atmosphere. Baby hamster kidney-21 (BHK-21) cells were maintained in MEM supplemented with 8% FBS, high glucose (4 g/L), penicillin (50 U/mL), and streptomycin (50 mg/mL) at 37°C in a 5% CO2-humidified atmosphere.

### Mice

The animal experiment was conducted in accordance with the Official Mexican Standard Guidelines for the Production, Care, and Use of Laboratory Animals (NOM-062-ZOO-1999). Neonatal CD-1 (ICR-CD1 strain 022) and adult AG129 (IFN α/β/γ R^−/−^ strain 129/Sv) mice were maintained under specific-pathogen-free conditions at the Laboratory Animal Production and Experimentation Unit (UPEAL-CINVESTAV). Protocols 0382–24 (for CD-1 mice) and 048–02 (for AG129 mice) were approved by the Animal Care and Use Committee (CICUAL) at CINVESTAV-IPN, Mexico. Charles River Laboratories originally provided CD-1 mice. Marshall Bioresources initially provided AG129 mice.

## Method details

### Depletion of extracellular vesicles from fetal bovine serum

The fetal bovine serum (FBS) (Cat. 16000044; Gibco) was inactivated and ultracentrifuged at 100,000 × g for 18 h at 4°C (Type 55.2 Ti rotor in a Beckman Optima™ L-60 Ultracentrifuge). The supernatant (SN) was filtered through a 0.22 μm Millipore membrane (Cat. GSWP04700, Merck Millipore) to remove EVs, yielding EV-depleted FBS.

### Cell culture and viral strain

In our study, we used one mosquito and two mammalian cell lines. *Aedes albopictus* larvae clone C6/36 HT (C6/36) [12,13] cells were cultured in Minimal Essential Medium (MEM) (Invitrogen) supplemented with 10% EVs-depleted FBS, vitamins (Invitrogen), 0.034% sodium bicarbonate (J. T. Baker), 100 μg/mL streptomycin, and 100 U/mL penicillin (Sigma). Cells were maintained at 32°C in a humidified atmosphere without CO2. The human hepatocellular carcinoma (Huh-7) cells were grown in advance Dulbecco's Modified Eagle Medium (DMEM) (complete medium) supplemented with 2 mM glutamine, 7% fetal bovine serum (FBS), high glucose (4 g/L), Amphotericin B (1000X), penicillin (50 U/mL), and streptomycin (50 μg/mL). Baby hamster kidney-21 (BHK-21) cells were maintained in MEM supplemented with 8% FBS, high glucose (4 g/L), penicillin (50 U/mL), and streptomycin (50 μg/mL). Both cells were maintained at 37°C in a 5% CO_2_ humidified atmosphere.

DENV serotype 2 (New Guinea C strain), kindly donated by the Instituto de Diagnóstico y Referencia Epidemiológicos. DENV was propagated three times in C6/36 cells (DENV2-infected) as described previously [14]. DENV was propagated in C6/36 cells grown to 80% confluence in a T-75 flask. Absorption of the inoculum was performed in MEM medium without FBS at a 1:70 ratio of DENV to cells for 2 h under constant agitation (CA). Subsequently, complete medium was added, and the culture was left for 5 days post-infection (dpi). The supernatant was centrifuged and filtered through a 0.22 μm Millipore membrane filter (Merck Millipore) to obtain DENV2. Uninfected C6/36 cells were used as a control (Mock-treated). The viral yield was determined by plaque assay in BHK-21 cells [15].

Huh-7 cells seeded at 80% of confluence were infected with DENV2 at a multiplicity of infection of 0.001 in DMEM balanced salt solution for 2 h at 37°C. For infection with I-sEVs_AG_, the inoculum was incubated for 12 h at 37°C. Subsequently, in both cases, infection was allowed to continue for 48 h post-infection (hpi).

## Transmission electron microscopy

The C6/36 cells grown in T-75 flasks (Corning) were Mock-treated or DENV-infected at an MOI of 0.001–48 hpi. Then, the cells were collected by scraping and fixed with 2.5% glutaraldehyde in 0.1 M sodium cacodylate buffer (pH 7.2) for 1 h at room temperature (RT), followed by post-fixation with 1% osmium tetroxide for 1 h at RT. The samples were dehydrated through an ethanol gradient and propylene oxide, and then embedded in Polybed epoxy resin and polymerized at 60 °C for 24 h. Finally, 70-nm-thick sections were stained with uranyl acetate and lead citrate and analyzed using a JEOL JEM-1011 transmission electron microscope (JEOL Ltd., Tokyo, Japan).

## Isolation of small extracellular vesicles from the cell culture supernatant

The C6/36 cells were seeded at 80% in T-175 flasks (Corning). Cells were DENV-infected with an adsorption medium prepared with MEM medium at an MOI of 0.001 for 2 h at 35°C under CA. Then, the cells were washed once with sterile 1X phosphate-buffered saline (PBS) and complete with MEM supplemented with 10% EVS-depleted FBS. The supernatants (SNs) from MOCK-treated and DENV-infected C6/36 cells were collected after 48 hpi and centrifuged using a Sorvall H-400 centrifuge at 900 × g to remove viable cells and 2,000 × g for 10 min to remove debris. The supernatants were collected and centrifuged at 10,000 × g for 1 h at 4°C (Beckman Coulter Avanti® J-26 XP centrifuge/JA-25.50 rotor). The pellets were resuspended in 200 µL of 1X PBS, yielding (lEVs). The SNs were filtered through a 0.22 μm Millipore membrane filter (Merck Millipore). Then, the clarified SNs were recovered and ultracentrifuged at 120,000 × g for 80 min at 4°C with a Type 55.2 Ti rotor in a Beckman Optima™ L-60 Ultracentrifuge. The SNs were removed as thoroughly as possible, and the pellets were resuspended in 200 μL of 1X PBS and pooled, obtaining small extracellular vesicles (sEVs). The SNs were again ultracentrifuged at 120,000 × g for 80 min at 4°C. This step was repeated twice. Finally, the sEVs were resuspended in 500 µL 1X PBS. It should be noted that the 1X PBS used for sEV isolation was ultracentrifuged for 18 h at 100,000xg at 4°C and then filtered through a 0.22 μm Millipore membrane filter (Merck Millipore) before use. Immediately, the sEVs were treated with Total Exosome Isolation Reagent (from cell culture media) (Cat. 4478359; Invitrogen) according to the manufacturer's instructions. This step helps us separate sEVs from water, allowing us to concentrate them intact and enrich the vesicles obtained. The mixture of sEVs and reagent was incubated overnight at 4°C and then centrifuged at 10,000 × g for 1 h at 4°C. The sEVs eluted from Non-Infected (NI-sEVs) and Infected cells (I-sEVs) were collected in a clean tube. The NI-sEV was used as a control. The NI – and I-sEVs were stored at – 80°C until use.

## Negative staining electron microscopy analysis

The purified sEVs were placed on the surface of the formvar-coated copper grids and stained with 2.5% uranyl acetate. The grids were allowed to dry, coated with carbon, and examined through a JEM-1011 transmission electron microscope.

## Immunoblotting analysis

A Pierce BCA protein assay kit (Cat. 23225; Thermo Scientific) measured protein concentrations. Polyacrylamide gradient gel electrophoresis (15%) in denaturing conditions (sodium dodecyl sulfate-polyacrylamide gel electrophoresis [SDS-PAGE]). Total cell and sEVs lysates (30 μg), or total lysates from brain tissue CD-1 and AG129 mice (50 μg) were separated by protein per lane and transferred thereafter to nitrocellulose membranes (Bio-Rad) and blocked with 10% nonfat milk in PBST (1X PBS/0.01% Triton X-100) for 1 h at RT. As a positive control for the sEVs proteins, C6/36 cells were used. The C6/36 cells, grown at 60% or 70% confluence, were either Mock-Treated or DENV2-infected at an MOI of 0.001 for 48 h post-infection. Then, the adherent C6/36 cells were washed 3 times using cold 1X PBS and were all resuspended in RIPA buffer [0.1% SDS, 0.5% NP40, 10 mM Tris-Cl pH 7.5, 1 mm EDTA, 150 mM NaCl, 0.5% deoxycholate plus protease inhibitor cocktail (Complete, Sigma-Aldrich)]. The lysate of sEVs proteins was obtained using the RIPA buffer. All antibodies to the tetraspanin proteins (Santa Cruz Biotechnology) were used at 1:10,000. The immunoblotting was performed to detect human CD9 (Cat. sc-59140; RRID: AB_1120766), human CD63 (Cat. sc-365604; RRID: AB_10847220), human CD81 (Cat. sc-166029; RRID: AB_2275892), or human Annexin V (ANX) (Cat. sc-74438; RRID: AB_1118989). All antibodies to viral proteins (Genetex) were used at a 1:5,000 dilution. The immunoblotting was performed to detect Rabbit Capsid (C; Cat. GTX103343; RRID: AB_1240697), and rabbit non-structural protein 1 (NS1), 3 (NS3; Cat. GTX124252; RRID: AB_11171668), and 5 (NS5; Cat. GTX124253; RRID: AB_11169932). Mouse Actin (Cat. sc-81178; RRID: AB_2223230) was used as a loading control. HRP-conjugated goat anti-mouse or anti-rabbit IgG antibodies (1:5,000; Cell Signaling) with 5% nonfat milk in PBST were used as secondary antibodies. The proteins were visualized using the Super Signal West Femto Chemiluminescent Substrate (Cat. 34096; Thermo Scientific). Digital images were obtained using ImageQuant LAS 4000 System (GE Healthcare) and analyzed with ImageJ software.

## Amplification of complete DENV genome

The cDNA generated from total RNA isolated from C6/36 cell-derived I-sEVs was used as a template for amplifying the DENV2 genome. DENV viral stock and NI-sEV were used as positive and negative controls. The total RNA was extracted from 30 µg of sEVs using TRIzol reagent (Cat. 15596026; Invitrogen) according to the manufacturer's specifications. The cDNA was quantified and amplified using the ImProm-II™ Reverse Transcriptase protocol (Cat. A3803; Promega). The oligonucleotides used for amplification are shown in table supplementary 1 (Table S1). We amplified fragments 1–11 of variable sizes by regular PCR. Using the recombinant polymerase (Cat. 10342046; Thermo Scientific), regular PCR was performed under the following conditions: initial denaturation at 95°C for 5 min, followed by 40 cycles of steps consisting of 95°C for 30 s, 58°C for 30 s, and 72°C for 1 min. Fragments 1-11, amplified products of the following sizes: 1,103 bp; 1,010 bp; 1,124 bp; 1,114 bp; 1,118 bp; 1,141 bp; 1,104 bp; 1,135 bp; 1,110bp; 1,048 bp; and 717 bp, respectively (Table S1). The PCR reactions were subsequently run on 1% agarose gels, imaged with the SYBR® Safe DNA Gel Stain (Cat. S33102; Invitrogen), and processed using Image Lab software from the manufacturer (Bio-Rad).

## Capture assay

A capture assay was conducted to evaluate the ability of mosquito-derived sEVs to be internalized by Huh-7 cells. The sEVs membrane was labeled with the lipophilic dye DiL (Cat. D282; Invitrogen), while the RNA content was stained using SYTO™ RNASelect™ (Cat. S32703; Invitrogen). NI-sEVs were resuspended in 100 µL of 1X PBS. Sequential staining was performed, beginning with the SYTO™ RNA Select™ labeling solution according to the manufacturer’s protocol, incubating for 30 min at 37°C under constant agitation. Subsequently, the membrane of NI-sEVs^SYTO^ was counterstained with DiL (5 µg/mL) for 20 min at RT under constant agitation. 2% 1X PBS-albumin was added to remove DiL excess and incubated for 10 min at RT under constant agitation. Following the manufacturer's instructions, the stained NI-sEVs^SYTO/DIL^ were concentrated using the Total Exosome Isolation Reagent (Invitrogen) to isolate intact labeled sEVs. The sample was incubated overnight at 4°C, followed by centrifugation at 10,000 × g for one hour at 4°C. For the interaction assay, 30 µg of NI-sEVs^SYTO/DIL^ protein was incubated with Huh-7 cells, either plated on slides or in 24-well plates, at 70-80% confluency. Cell interaction was homogenized by placing the cells at 4°C for 30 min, then switching to 37°C for the following times: 0, 5, 15, and 30 min post-inoculation (mpic); and 1.5, 3, and 12 h post-inoculation (hpic).

## Viral inactivation using acid glycine

To inactivate free viral particles and prevent infection *in vitro* and *in vivo* assays, acid glycine (AG) treatment was used. Using an MOI of 0.001 for DENV2, a volume ratio of 2:1 between the initial volume of DENV2 and AG (0.1 M, pH 2.2) was used and incubated for 15 min at RT, stabilizing the pH with Tris-EDTA buffer (TE) (pH 7.6, 1 M), again using a 2:1 volume ratio to the initial volume and TE; and incubated for 15 min at RT, obtaining the AG-inactivated condition (DENV2_AG_). It is then incubated as an adsorption inoculum for 2 h, after which the adsorption medium is removed, and infection is allowed to proceed for 48 hpi. For *in vitro* infection, 30 µg of AG-inactivated I-sEVs protein (I-sEVs_AG_) was used, inactivated as mentioned above. DENV2 was used as a positive control. Cells infected with NI-sEVs and DENV2_AG_ were used as controls. For *in vivo* infections, 30 µg of protein and different concentrations of I-sEVs_AG_ protein were used for neonatal CD-1 mice, and 40, 70, and 100 µg of I-sEVs_AG_ protein were used for adult AG129 mice.

## Confocal microscopy

Huh7 cells grown at 70-80% confluence on coverslips placed in 24-well plates were infected with I-sEVs_AG_ for 48 hpi. Additionally, brain tissue sections from AG129 mice infected with I-sEVs_AG_ were prepared. The tissue from the mice was frozen until the mice were euthanized. Frozen tissue sections were prepared at a thickness of 0.8 µm.

The Huh-7 infected cells, from capture assays or tissue sections of AG129 mice, were washed three times with 1X PBS and fixed with 4% paraformaldehyde (PFA) for 30 min at 4°C. A permeable solution (0.2% saponin, 1% FBS, and 1X PBS) was used to permeabilize cells for 30 min at RT. Later, they were incubated overnight at 4°C to detect rabbit polyclonal NS3 antibody (1:200; Cat. GTX124252; Genetex), rabbit NS5 (1:200; Cat. GTX124253; Genetex), or mouse prM/E (2H2) (1:50; Cat. D3-2H2-9-21; ATCC). As secondary antibodies, goat anti-mouse Alexa Fluor 488 (Cat. A21202; Invitrogen) and goat anti-rabbit Alexa Fluor 555 (Cat. A21428; Invitrogen) were used, and the nuclei were counterstained with Hoechst dye (Cat. sc-394039; Santa Cruz Biotechnology). The slides were observed on a Leica TCS SP8 Confocal Microscope (Leica Microsystems), and the images were analyzed using the Leica Application Suite X Core Offline v3.3.0.

## Flow cytometry assay

Huh-7 cells infected with I-sEVs_AG_ were analyzed to determine the percentage of infected cells. The antibody prM/E (ATCC) was used to determine the percentage of infected cells by flow cytometry. A goat anti-mouse Alexa Fluor 488 secondary antibody (Cat. A21202; Invitrogen) was used. In addition, capture assays quantify the uptake of labeled sEVs by Huh-7 cells, determining the positive DiL-labeling. The flow cytometry assay was performed on a BD LSR Fortessa, and the data were analyzed using FlowJo v. 10. Three independent experiments, each performed in duplicate, were conducted to determine the percentage of infected cells.

## Plaque assay

Supernatants from the DENV-2-infected untreated and treated cells were used to determine the viral yield using the plaque-forming units (PFU) modified assay of Morens et al. [15]. Using an initial volume of 50 µL of SN or homogenate from infected AG129 mouse brain tissue, serial dilutions were performed and plated on a monolayer of BHK-21 cells at 100% confluence. After two h incubation, each well received 500 µL of 0.8% medium viscosity carboxymethylcellulose sodium salt (CMC) (Cat. C4888; Sigma-Aldrich) [0.8% of CMC, 2x Earle´s minimum essential (MEM) (Cat. 11935-046; Gibco) with 10% heat-inactivated fetal bovine serum, 1% glutamine 2 mM and 100 U of penicillin plus 100 ug/ml streptomycin] was added, allowing for 5 dpi at 37°C with 5% CO_2_. The supernatant was then removed, and the adherent cells were fixed and stained with Naftol Blue Black (NBB) (Cat. N3393; Sigma-Aldrich) [0.1% naphthol blue–black, 6% glacial acetic acid, and 1.36% sodium acetate]. Three independent experiments, each performed in duplicate, were conducted for each assay.

## CD-1 mouse DENV infection

For *in vivo* DENV infection mediated by sEVs, we used the protocol described by Cole et al. [16]. Two-day-old neonatal mice were intracranially (i.c.) inoculated with 30 µg of sEVs. 1.25 × 10^6^ PFU/mL DENV2-infected was used as positive control. Mock-treated, NI-sEVs, and DENV2_AG_-infected mice were used as negative controls. All conditions were diluted in 20 μL of injectable water. All mice were euthanized and immediately frozen four days post-infection (dpi). For the following experiments, the mice were thawed, and their brains were removed to prepare for determining active replication by immunoblotting and viral genome quantification by qPCR-RT.

## AG129 mouse survival assays

For *in vivo* I-sEVs infection, 6-week-old mice per condition were intraperitoneally (i.p.) injected with 3 different concentrations (40, 70, and 100 µg/mL) of I-sEVs_AG_. 4 × 10^6^ PFU/mL of DENV2-infected cells was used as a positive control. Mock-treated, NI-sEVs, and DENV2_AG_-infected mice were used as negative controls. All conditions were diluted in 100 μL of injectable water.

The mice's weight, clinical signs, and survival of the disease were monitored daily until the day of euthanasia. The signs of the disease are based on a clinical score reported by Orozco et al. [17]. This table was updated according to the results obtained in this study to monitor the average morbidity of DENV-infected mice on a scale of 1–5, where “1” represents healthy and “5” moribund mice (Table 1). Two independent experiments were performed, each with 3 female and 3 male mice. Mice that died from causes other than infection were excluded. All mice were euthanized and immediately frozen. For the following experiments, the mice were thawed, and the brain was removed to prepare for determining active replication by immunoblotting, tissue immunolabelling, and section analysis by confocal microscopy, viral particle production by lytic plaque assays, and viral genome quantification by qPCR-RT assays. The survival results, clinical scores, and mouse weights were plotted and analyzed using GraphPad Prism software version 6.0.

**Table 1.** New morbidity describes the clinical symptoms of AG129 mice infected with DENV and I-sEVs.

Score	Clinical signs of DENV disease
1	Healthy. No ruffled hair (no piloerection) or hunched posture. Normal ambulation and response to stimuli.
2	Increased mobility and response to stimuli. No hunched posture.
3	Sligh signs of lethargy, ruffled hair, and hunched posture. Head tilt changes, maintaining rotation to one side. It is possible to see the presence of closure (total or partial) of one eye (panuveitis) with the presence of grey material.
4	Increased lethargy, limited mobility, ruffled hair, hunched posture, and decreased response to stimulation may be present. Paralysis of the hind limbs may also be present.
5	Moribund, ruffled, hunched posture with reduced or minimal mobility. Significant reduction in body weight (20–30% of total weight) consistent with inability to obtain food and water. Immediate euthanasia.

This work proposes a new morbidity scale describing symptoms in AG129 mice infected with DENV. The clinical symptoms were first described by Orozco et al. (2012) but are complemented by new symptoms described in this paper.

## Viral yield from brain tissue of AG129 mice

The viral particles from AG129 mice were produced from 100 mg of brain tissue. The tissue was resuspended in 200 μL of Hank's medium (Cat. 14170120; Thermo Scientific) and macerated at medium speed using a sterile tip with a tissue homogenizer (Cat. 099C k54; Glas-Col.). Add another 200 μL of Hank´s medium and pass through a 23-gauge needle 5 times. The sample was centrifuged at 21,100 × g at 4 °C for 15 min, and the supernatant was filtered through a 0.22 μm Millipore membrane filter (Merck Millipore). The lytic plate assay technique described above is performed.

## Viral RNA from brain tissue CD-1 and ag129 mice

Brains from CD-1 and AG129 mice infected with I-sEVs_AG_ were used to measure viral load. The total RNA was extracted from 100 mg of brain tissue from 2 or 3 mice per condition (each mouse was considered a single experimental unit) using TRIzol reagent (Cat. 15596026; Invitrogen) according to the manufacturer's instructions. Homogenization was carried out using a TissueRuptor (Qiagen) at medium speed. All samples were treated with DNase I (Cat. M0303; BioLabs) according to the manufacturer's instructions. 1 mg total RNA was reverse transcribed to cDNA using the ImProm-II™ Reverse Transcriptase protocol (Cat. A3803, Promega). Finally, from 50 and 200 ng of cDNA (for CD-1 and AG129 mice, respectively), quantitative polymerase chain reaction (qPCR) was performed to quantify DENV-2 viral RNA copies from mouse brains, using the following primers: Fw: 50-CAATATGCTGAAACGCGAGA-30; Rv: 50-TGCTGTTGGTGGGATTGTTA-30 (Table S1) [18]. For each PCR reaction, 5 µl of SYBR Green PCR-Master mix (iTaq Universal SYBR Green One-Step Kit, BIO-RAD Cat. 172-5150), 0.5 µl of each primer 10 mM, and 1 µl of cDNA diluted in RNase-free water were added. Thermal cycling conditions were as follows: an initial step at 50°C for 2 min, followed by 95°C for 1 min, and then 40 cycles of 95°C for 10 sec and 60°C for 30 sec, using the ECO Illumina System. In parallel, we generated a standard curve (10^8^, 10^7^, 10^6^, 10^5^, 10^4^, 10^3^, 10^2^) from a plasmid containing a 151 bp insert of the DENV-2 genome (NCBI ID: NC_001474.2) corresponding to the C protein region [19], which was amplified with the same primers mentioned above. The threshold was adjusted with a Mock-treated mouse sample and the non-templated control. The results were analyzed using EcoStudy software version 5.04890. Data were expressed as viral copies in 100 mg of tissue.

## Protein extraction from brain tissue of CD-1 and AG129 mice

Viral infection in the brains of CD-1 and AG129 mice infected with I-sEVs_AG_ was confirmed by immunoblotting. Total protein extraction from the brain tissue of CD-1 and AG129 mice infected with I-sEVs_AG_ was performed using 100 mg of tissue. The tissue was homogenized and resuspended in 200 µL of RIPA buffer. Homogenization was carried out using a VCX130-220 V ultrasonic processor (Sonics Materials™) at 80% power intensity, with a total sonication time of 1 min divided into 10-second pulses interspersed with 15-second rest intervals to prevent overheating. Following sonication, protein concentration was quantified using the Pierce BCA Protein Assay Kit (Cat. 23225; Thermo Scientific), according to the manufacturer’s protocol. The Immunoblotting technique described above is performed.

## Brain tissue sections from AG129 male mice

The extracted brains were washed in 1x PBS. They were fixed with 4% paraformaldehyde overnight at 4°C. They were then immersed in 10% sucrose overnight at 4°C. Brains were frozen and protected with Tissue Freezing Medium (Cat. 14020108926; Leica), and 0.8 µm sections were cut on a cryostat and placed on slides. The brain tissue sections were permeabilized with a permeabilization solution for 4 h. Tissue sections were analyzed to detect rabbit polyclonal NS3 (1:200; Cat. GTX124252; Genetex), and rabbit NS5 (1:300; Cat. GTX124253; Genetex). The confocal microscopy technique described above is performed.

### Statistical analysis

The statistical significance of the difference observed between data sets was analyzed using GraphPad Prism 8.0. The non-paired, two-tailed Student t-test was performed (for data to compare two means) and one-way ANOVA with Tukey multiple comparisons post hoc test (for data to compare three or more means). For the in vivo assays, the Kaplan – Meier survival curves were drawn. The Wilcoxon and Mantel–Cox tests were used to compare survival rates between infected groups in GraphPad Prism 8.0. Error bars represent the standard error of the mean (SEM) values. In all cases, a *p* < 0.05 was considered statistically significant.

## Results

### Characterization of mosquito cell-Derived sEVs.

We utilized *Aedes albopictus* cell lines (C6/36) as a model to investigate the interactions between dengue virus serotype 2 (DENV2) and mosquito vectors. Specifically, we analyzed whether the cells secrete small extracellular vesicles (sEVs) and whether these are involved in the transmission of pathogens. The presence of multivesicular bodies (MVB) within intraluminal vesicles (ILV) is primarily responsible for generating sEVs. First, we analyzed the presence of MVB and ILV in non-infected (NI) and DENV2-infected (I) mosquito C6/36 cells. Our results showed that at a multiplicity of infection (MOI) of 0.001, DENV2 significantly increased the number of infected C6/36 cells at 48 h post-infection (hpi), preventing the formation of cell syncytia (Fig. S1).

**Figure 1.**
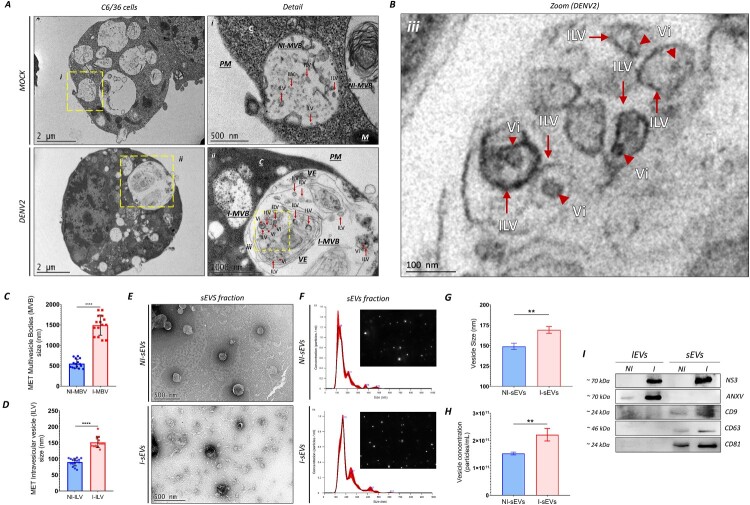
Mock-treated and DENV2-infected C6/36 cells secrete sEVs with different diameters and concentrations. (A) Transmission Electron Microscopy (TEM) analysis reveals sEVs secreted by both Mock-treated and DENV2-infected cells at 48 h post-infection (hpi). (B) Close-up TEM analysis of DENV2-infected cells shows virus-like particles (Vi, tip) within intraluminal vesicles (ILVs, arrow). Key structures are labeled: multivesicular body (MVB), ILV, mitochondria (M), double-membrane vesicles (Ve), membrane packets (Vp), and Vi. C-D) TEM analysis reveals an increase in size in I-MVB (C) and I-ILV (D), compared to NI-MVB and NI-ILV, respectively. Each point represents the size of a vesicular structure (MVBs or ILVs, respectively) in the cell interior. (E) NI-sEVs and I-sEVs released from C6/36 cells were isolated by ultracentrifugation and visualized using negative staining electron microscopy. Both sEVs fractions exhibit a spherical structure with a characteristic “cup-like” morphology, consistent with sEVs. (F) Histogram of nanoparticle tracking analysis (NTA) analysis of NI-sEVs and I-sEVs samples. Samples were diluted at 1:100 and 1:200 for the NI-sEVs and I-sEVs conditions, respectively. (G-H) NTA analysis demonstrates that I-sEVs exhibit an increase in vesicle diameter (G), and quantity (H) compared to NI-sEVs. Data were represented as mean ± standard error of the mean (SEM). Two-tailed unpaired Student´s T-Test performed statistical comparison. **p < 0.0055. (I) Immunoblot analysis confirms the purity of NI-sEVs and I-sEVs using human antibodies against CD9, CD63, and CD81 sEVs markers. The lEVs sample, with its annexin V (ANXV) marker, served as a control. NI-sEVs and I-sEVs show the presence of CD9, CD63, and CD81 markers, while ANXV is absent, confirming the purity of sEVs.

Electron microscopic analysis (Figure 1A) revealed the presence of ILVs within MVBs located at the cell periphery near the plasma membrane in the Non-Infected (NI-ILV and NI-MVB) and DENV2-Infected (I-ILV and I-MVB) conditions, respectively. Notably, electron-dense points of a size comparable to viral-like particles (Vi) were visualized within I-ILVs (Figure 1B) but were absent in NI-ILVs. I-MVBs exhibited a significant increase in diameter (approximately 1,495.3 nm ± 247.9) compared to NI-MVBs (approximately 551.7 nm ± 109.8) (Figure 1C). Additionally, a higher number of membranous bodies were observed in I-MVBs than in NI-MVBs. I-ILVs were also more prominent (approximately 151.7 nm ± 16.5) than NI-ILVs (approximately 90.3 nm ± 11.9) (Figure 1D). These findings demonstrate that mosquito cells can generate primordial vesicle membranous bodies (MVB and ILV) that facilitate the production of sEVs under both conditions. However, in DENV2-infected cells, there is a marked increase in the number and size of MVB and ILV.

The next step was to analyze whether C6/36 cells can secrete sEVs and whether mosquito-borne Orthoflaviviruses utilize them as a mode of pathogen transmission. The NI– and I-C6/36 cell supernatants were subjected to consecutive centrifugations to separate viable cells, cell debris, and large extracellular vesicles (lEVs). Transmission electron microscopy (TEM) assays performed on fractions of sEVs derived from C6/36 cells revealed NI – and I-sEVs vesicles. The isolated sEVs displayed intact vesicles with a cup-holder shape with typical morphological characteristics (Figure 1E). The vesicular population of NI-sEVs had a homogeneous population with a smooth and uniform surface. In contrast, I-sEVs showed a more heterogeneous vesicle mixture with irregular exteriors. Indirect analysis showed that NI-sEVs were smaller in diameter than I-sEVs, and vesicular populations with larger diameters (such as lEVs) were not observed. Quantitative analysis of the vesicular population was performed using nanoparticle tracking analysis (NTA) to determine diameter and concentration. Results showed that, despite NI – and I-sEVs following a similar diameter distribution of sEVs (less than 200 nm), significant differences were found between groups in diameter and concentration. The NI – and I-sEV histograms show a distinct peak between 100 and 200 nm (Figure 1F), with discrete peaks at larger sizes. The histogram of NI-sEVs shows more discrete peaks at larger sizes than the more prominent peaks in the histogram of I-sEVs, corroborating the homogeneity of the NI-sEV population. When comparing sEVs numbers, we determined that I-sEVs increased in diameter (170.2 nm ± 10.4 vs. 149.1 nm ± 9.4) (Figure 1G) and concentration (2.22 × 10^11^ vs. 1.53 × 10^11^) (Figure 1H) compared to NI-sEVs, respectively, indicating that sEVs diameter and concentration increase with viral infection. The lEVs are the most abundant vesicle populations isolated during the separation of sEVs and exhibit specific cell markers, such as Annexin V (ANXV). To confirm the purity of the vesicle population, we performed immunoblot analysis for specific sEVs biomarkers, including tetraspanins (CD9, CD63, and CD81). Annexin and tetraspanin markers have been described by the ISEV as exclusive markers of lEVs and sEVs, respectively [20]. We observed the detection of all tetraspanins in the sEV vesicular population, both in NI-sEV and I-sEV, but not in the NI – and I-lEV populations. We observed ANXV protein in the NI – and I-lEV vesicular population but not in the NI – or I-sEVs population (Figure 1I). This result indicates that our sEV population is free of other vesicular populations. Both results demonstrated that our EV isolation protocol efficiently isolates sEVs, removing larger particles and other-sized vesicles from the supernatant.

### Mosquito-derived I-sEVs contain viral components.

**Figure 2.**
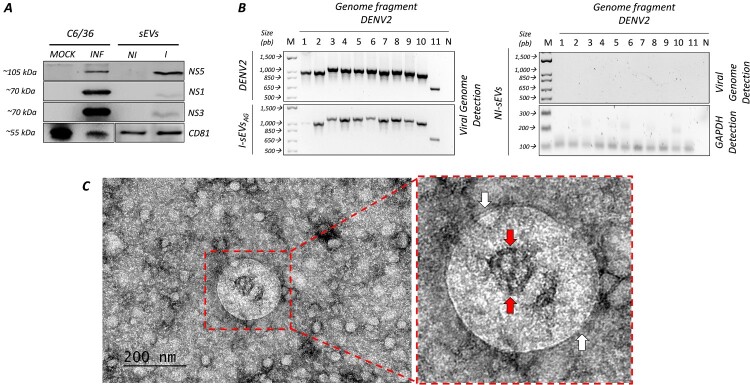
Mosquito cell-derived I-sEVs contain viral non-structural (NS) proteins, full-length DENV2 genome, and particles with sizes similar to double-membrane viruses. (A) Immunoblot analysis confirms the presence of DENV2 NS1, NS3, and NS5 proteins, along with the tetraspanin CD81 (sEVs marker), in the I-sEVs sample. Total cell lysates from Mock-treated (Mock) and DENV2-infected (INF) C6/36 cells (MOI 0.001, 48 h post-infection (hpi)) were used as controls. I-sEVs (I) exhibit all three viral proteins, with a pronounced increase in NS5 levels. No viral proteins were detected in Mock-treated or NI-sEVs (NI) conditions. DENV-specific antibodies against NS1, NS3, and NS5, as well as human anti-CD81, were used for detection. CD81 was detected in all conditions, confirming the presence of sEVs. Due to photobleaching in the immunoblot, a lane for the CD81 protein was cut between the C6/36 and sEV conditions. (B) Agarose gel electrophoresis demonstrates the amplification of overlapping fragments representing the full-length DENV2 genome in the I-sEVs sample. cDNA was synthesized from the total RNA of NI-sEVs and I-sEVs, which served as templates for PCR amplification. Total RNA from DENV2-infected C6/36 cells (MOI 0.001, 48 hpi) was used as a control. Eleven fragments (fragment sizes detailed in Materials and Methods) were generated to confirm the presence of the complete DENV2 genome. GAPDH (110 bp) was amplified as a loading control for the NI-sEVs sample. N indicates a no-template control, and M represents the DNA marker, with selected marker bands shown for reference. The results are representative of three independent experiments. (C) Transmission electron microscopy (TEM) analysis reveals I-sEVsAG shows double-membrane vesicles inside them with sizes like those of viral particles (red arrow). These vesicles are observed alongside 30-60 nm vesicles, similar in size to viral particles inactivated by AG. I-sEVsAG were visualized by negative-staining electron microscopy. The complete figure's results represent three independent experiments.

A unique feature of sEVs is the ability to contain and transport biological material from the cells and the pathogens that infect them to target cells. sEVs can carry genetic and protein material, and the ability to carry viral components may give them an advantage in promoting new viral infections. Once the sEVs’ physical characteristics were determined, we assessed whether they contained pathogenic viral components, including viral proteins and a complete genome. The immunoblot analysis confirms the presence of DENV non-structural (NS) proteins in DENV-infected C6/36 and in 25 μg/mL of the total extract of I-sEVs (Figure 2A). We determined the presence of NS1, NS3, and NS5 on I-sEVs and DENV-infected mosquito cells. It was noted that NS1 and NS3 labeling decreased compared to total cell lysates at 48 hpi. Interestingly, we not only detected the presence of the NS5 protein, which plays a crucial role in replicating the viral genome, but also observed enhanced NS5 viral protein labeling in I-sEVs compared to DENV-infected C6/36 cells. As expected, these proteins were undetected in MOCK-infected C6/36 cells and NI-sEVs negative controls. Human tetraspanin CD81 was used as a marker for sEVs.

The next step was determining whether mosquito I-sEVs could carry the complete DENV2 genome. PCR analyses were performed using oligonucleotides used by Vora et al. [8]. These oligonucleotides generate amplicons that overlap to amplify the entire DENV2 genome. The characteristics of fragments 1–11 are shown in Table S1. We included DENV2 laboratory virus stock as a positive control. Agarose gel electrophoresis demonstrates the amplification of overlapping fragments representing the full-length DENV2 genome in the I-sEVs sample (Figure 2B). Although we found the presence of the complete DENV genome associated with the I-sEVs, we could determine that there was a decrease in the amount of all the fragments evaluated. The fragments that flank the genome (5' – and 3'-non-coding regions) have a more significant decrease in the viral stock control. Finally, negative-staining electron microscopy reveals that double-membrane I-sEVs contain virus-like particles with a double membrane (Figure 2C). These findings collectively demonstrate that I-sEVs are associated with viral components that can promote a new infection.

### Huh-7 mammalian cells take up and support viral infection via I-sEVs

After determining the physical characteristics of the sEVs, we assessed their ability to promote infection in human hepatocarcinoma (Huh-7) cells. However, to carry out the infection, sEVs from mosquito cells must be biocompatible with mammalian cells. Therefore, we decided to study the ability of Huh-7 cells to capture sEVs released by mosquito cells. With the NI-sEVs condition, it was decided to double-stain with the lipophilic tracker dialkyl carbocyanine (DiL) dye (Invitrogen™ D3911), labeling the vesicular membrane. At the same time, the RNA content was counterstained with SYTO-RNASelect dye (SYTO RNA) (Invitrogen™ S32703), resulting in double-labeled sEVs (NI-sEV^Dil/SYTO^). Using interaction kinetics, we employed confocal microscopy and flow cytometry assays to observe that the Huh-7 cells were unaffected (Fig. S2). The intake of sEVs by Huh-7 cells was validated using NI-sEVs^Dil/SYTO^.

As evidenced by DiL/SYTO RNA immunofluorescent labeling, sEVs entry is not a passive process because the interaction between NI-sEVs^Dil/SYTO^ and the surface of Huh-7 cells happens after 15 min post-interaction (mpit) and increases at 1.5 h post-interaction (hpit). We observed uptake at 3 hpi, with complete cell labeling at 12 hpi. Interestingly, SYTO-RNASelect labeling can only be observed until further accumulation of NI-sEVs^Dil/SYTO^ in 3 and 12 hpit. The labeling of both DiL and SYTO-RNA is only observed to be widely distributed in the cytoplasm, and at no time were labelings found inside the nucleus (Fig. S2A). Quantification of the number of DiL-positive cells by flow cytometry assays confirms that at the early times of 0, 5, and 15 mpit, the DiL-positive population is less than 1%, increasing to 2.13% at 1.5 hpit, 6.29% at 3 hpit, reaching a maximum value of 36% at 12 hpit (Fig. S2B). These results confirm biocompatibility and capture of mosquito-derived sEVs with human Huh-7 cells.

To investigate whether viral components (Dengue RNA, viral genome, or viral particles) contained in mosquito cell-derived sEVs are viable and capable of infecting vertebrate host cells to form infectious particles, we utilized NI– and I-sEVs from C6/36 cells at 48 hpi. To assess the total infectivity of the I-sEV-enriched vesicle population, we first ensured the complete inactivation of free viral particles that could be present in the preparation using an acidic glycine (AG) wash (pH 3) followed by neutralization with TRIS-HCL (1M pH 7.6) (Fig. S3A). To confirm the efficacy of AG treatment, we tested whether AG could completely inactivate the DENV2 viral stock. DENV2 was treated with (DENV2_AG_) or without (DENV2) AG wash and used to infect mammalian Huh-7 cells at an MOI of 0.001, allowed to proceed for 48 hpi. Using confocal microscopy, we detected the presence of structural pre-membrane (prM/E) and NS3 viral proteins exclusively in DENV2-infected cells. In contrast, no viral protein labeling was observed in DENV2_AG_-infected cells (Fig. S3B). Flow cytometry analysis showed an increase in DENV2 infection, whereas no active infection was detected in DENV2_AG_-infected cells (Fig. S3C). Furthermore, DENV2 infection produced approximately 1 × 10^7^ viral particles, whereas no active viral particles were detected in DENV2_AG_ (Fig. S3D). These results demonstrate that AG washing effectively inactivates free DENV2 viral particles.

**Figure 3.**
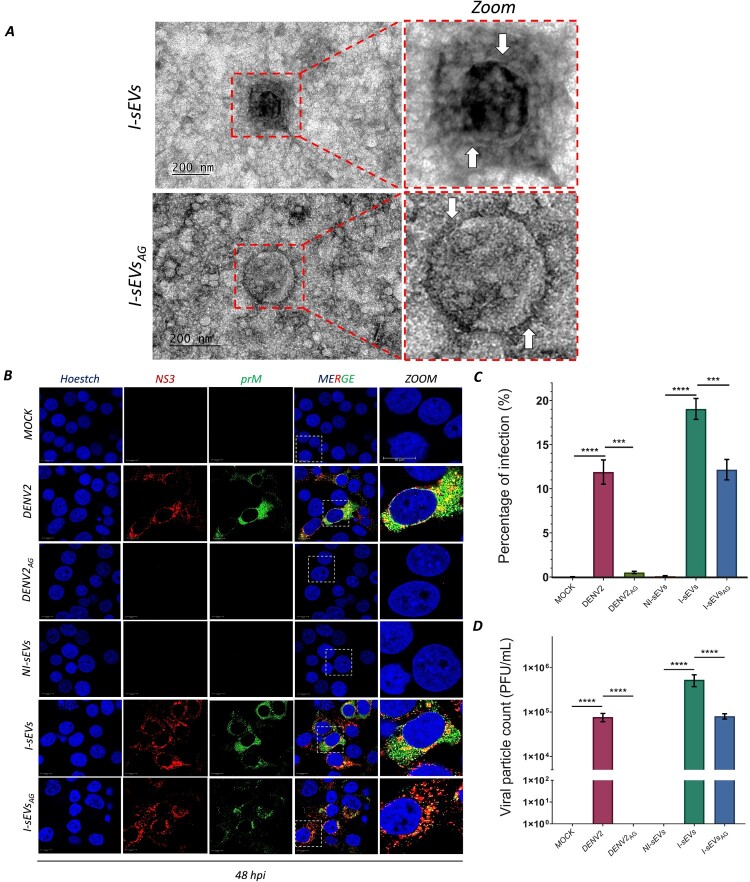
I-sEVs_AG_ facilitates viral infection and increases viral progeny in mammalian Huh-7 cells. (A) Transmission electron microscopy (TEM) analysis reveals that the structure and morphology of I-sEVs are not affected after treatment with AG. I-sEVs has a double membrane (indicated by the white arrow) that is maintained in after AG (I-sEVs_AG_). (B) Immunofluorescence analysis demonstrates increased viral infection in Huh-7 cells treated with 30 µg/mL I-sEVs_AG_ at 48 hpi. Mock-treated, DENV-, and DENV2_AG_-infected Huh-7 cells served as controls. I-sEVs_AG_ did not suppress viral infection compared to DENV2_AG_. Viral proteins were detected using DENV-specific antibodies against NS3 coupled to anti-rabbit Alexa-555 (red) and prM/E coupled to anti-mouse Alexa-488 (green). Nuclei were counterstained with Hoechst (blue). The last column shows a close-up view of individual cells for each condition, with a 10 µm scale bar. (C) Flow cytometry analysis confirms increased viral infection in Huh-7 cells treated with I-sEVs_AG_ at 48 hpi, quantified in fixed cells. Mock-treated, DENV-, and DENV2_AG_-infected Huh-7 cells were used as controls. I-sEVs_AG_ did not reduce viral infection compared to DENV2_AG_. The prM/E DENV antibody was used for detection, with a secondary antibody serving as an isotype control (data not shown). Data were represented as mean ± standard error of the mean (SEM). A one-way ANOVA was performed to conduct the statistical comparison, followed by Tukey post hoc tests. **p = 0.0030, ***p = 0.0001, ****p = <0.0001. (D) Lytic plaque assays reveal elevated viral progeny production in Huh-7 cells at 48 hpi, quantified from supernatants derived from I-sEVs_AG_-treated cells. Supernatants from Mock-treated, DENV-, and DENV2_AG_-infected Huh-7 cells were used as controls. I-sEVs_AG_ did not inhibit virion release compared to DENV2_AG_. No virion release was observed in Mock-treated, DENV2_AG_-, or NI-sEVs-infected supernatants. Data were represented as mean ± standard error of the mean (SEM). A one-way ANOVA was performed to compare the groups, followed by Tukey post hoc tests. ****p = <0.0001. The results shown in the complete figure represent three independent experiments.

After confirming that AG washing effectively inactivates free viral particles, we applied the AG wash to the total population of I-sEVs (I-sEVs_AG_) to evaluate whether their content could induce active infection. First, through negative staining TEM assays, we confirm that treatment with AG does not affect the morphology or integrity of I-sEVs (Figure 3A). For the infection assays, a total protein concentration of 30 µg/mL of NI-sEVs and I-sEVs_AG_ was used. Mock-treated, DENV2– and DENV2_AG_-infected MOIs of 0.001 were used as negative and positive controls, respectively. Interaction was allowed for 12 h, and infection was assessed at 48 hpi in Huh-7 cells.

Immunofluorescence analysis demonstrates the presence of NS3 in the nucleus of Huh-7 cells infected with I-sEVs_AG_ at 24 hpi (Fig. S4), consistent with initial infection kinetics [21]. However, the presence of prM/E– and NS3-viral proteins significantly increased at 48 hpi (Figure 3B). We observed the presence of viral proteins in Huh-7 cells infected with DENV2 compared to those infected with DENV2_AG_, where the presence of these viral proteins was not observed. Interestingly, mosquito-derived I-sEVs or I-sEVs_AG_ could infect Huh-7 cells. However, we noted a decreased prM viral protein labeling in I-sEVs_AG_ compared to I-sEVs. As expected, no viral proteins were detected in the negative controls, including Mock-treated and NI-sEVs-infected cells. Flow cytometry evaluated prM viral protein labeling in infected cells (Figure 3C) and confirmed that no prM labeling was detected in cells infected with DENV2_AG_. In contrast, cells infected with DENV2 exhibited a significantly higher presence of prM viral protein at 48 hpi, with infection levels reaching 11.88%. Although an increase in the prM protein level was observed in I-sEVs-infected cells, reaching 19.04%, infection in I-sEVs_AG_ showed an infection level of 12.06%. Despite the reduction in infection levels in I-sEVs_AG_ compared to I-sEVs, it remained significantly higher than the Mock-treated and NI-sEVs-infected cells, where no viral proteins were detected. While cells infected with I-sEVs expressed viral proteins, we sought to determine whether these cells could produce viral particles independent of free viral particles (Figure 3D). Using supernatant from infected Huh-7 cells, we observed a significant difference in viral particle production. DENV2 infection produced 7.68 × 10^4^ PFU/mL compared with DENV2_AG,_ whereas no viral plaques were detected. For I-sEVs, viral particle production was observed in both conditions, with 5.31 × 10^5^ PFU/mL viral particles and 8.06 × 10^4^ PFU/mL for I-sEVs_AG_. Interestingly, although viral particle production was reduced in the I-sEVs_AG_ condition compared with the untreated preparation, there was no significant difference in viral particle production between DENV2 and I-sEVs_AG_. As expected, no viral plaques were detected in Mock-treated and NI-sEVs-infected cells. In summary, these results demonstrate that after inactivating free viral particles with AG, I-sEVs remains associated with viral factors that can promote viral infection.

### Neonatal CD-1 mice are susceptible to infection mediated by I-sEVs

**Figure 4.**
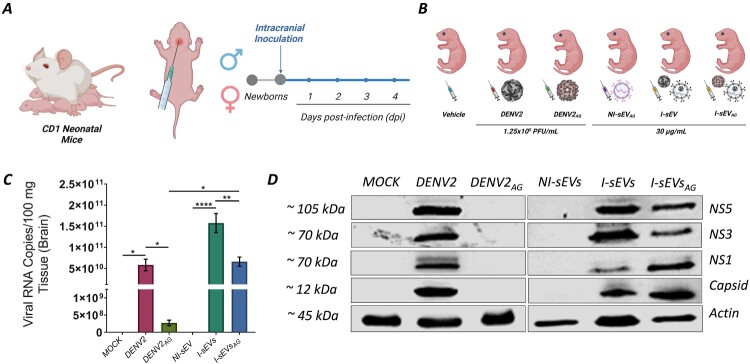
In vivo viral infection mediated by I-sEVs_AG_ in neonatal CD-1 mice. (A) Schematic representation of intracranial inoculations performed in neonatal CD-1 mice. Pups were injected with I-sEVs_AG_ on postnatal day 1 (P1), and infection was assessed 4 days post-infection (4 dpi). (B) Experimental groups included: (1) Mock-treated, (2) DENV-infected (1.25 × 10⁶ PFU/mL), (3) DENV_AG_-infected (1.25 × 10⁶ PFU/mL), (4) NI-sEVs_AG_-infected, (5) I-sEVs-infected, and 6) I-sEVs_AG_-infected. Inoculum from all conditions was diluted in 20 µL of injectable water. The graphics were elaborated using BioRender.com. (C) Quantification of viral RNA by real-time PCR revealed increased viral RNA levels in the brains of I-sEVs_AG_-infected mice. Although no infection was observed *in vitro* with DENV2_AG_, low levels of viral RNA were detected in group 3 (DENV_AG_-infected). Data were represented as mean ± standard error of the mean (SEM). A one-way ANOVA was performed to conduct the statistical comparison, followed by Tukey post-hoc tests. *p = 0.0332, **p = 0.0014, ***p = <0.0001. (D) Immunoblot analysis confirms the presence of DENV2 NS1, NS3, NS5, and structural capsid proteins in I-sEVs_AG_-treated brain lysates. Total brain lysates from Mock-treated, DENV2-infected, and DENV_AG_-infected neonatal CD-1 mice were used as controls. I-sEVs_AG_-infected samples exhibited all four viral proteins, while no viral proteins were detected in Mock-treated, DENV2AG-infected, or NI-sEVs-infected conditions. DENV-specific antibodies against NS1, NS3, NS5, and capsid were used for detection. Actin served as a loading control. The results shown in the complete figure represent two or three independent experiments.

I-sEVs_AG_ effectively infects Huh-7 cells *in vitro*; therefore, we sought to investigate whether this ability could happen in an *in vivo* model. In the initial studies, each two-day-old CD-1 mouse was intracerebrally (i.c.) inoculated with 30 µg/mL NI-sEVs and I-sEVs samples resuspended in 20 µL of injectable water. Four days post-infection (dpi), viral load and replication were analyzed using RT-qPCR and immunoblotting (Figure 4A). Mice were grouped into six categories (Figure 4B). The i.c. Inoculation of DENV2 in CD-1 pup mice resulted in 100% lethality between 8 and 10 days (data not shown). However, the mice were euthanized on the fourth dpi to compare viral replication. At this time, clinical signs with potential neurological involvement began to appear. The most evident neurological symptoms included difficulty turning over, imbalance, tremors, and claudication.

To identify viral replication, we determined the viral load in mice infected with I-sEVs_AG_ (Figure 4C). The viral load obtained by RT-qPCR assays indicates that mice infected with DENV2 exhibit a significant viral load of 5.84 × 10^10^ copies of viral RNA/100 mg. In the I-sEVs group, a significant increase in viral load was observed, with 1.57 × 10^11^ copies of viral RNA/100 mg, compared to the positive control. However, in the I-sEVs_AG_ group, viral replication was reduced to 6.64 × 10^10^ copies of viral RNA/100 mg. This is markedly different from the negative controls, including Mock-treated and NI-sEVs_AG_-infected mice, in which no viral load was detected. The DENV2_AG_ group detected 2.71 × 10^8^ copies of viral RNA/100 mg, likely attributable to residual inoculum in the inactivated viral particles. To further investigate replication, we analyzed the expression of NS-viral proteins, which are exclusively produced during active replication stages (Figure 4D). Viral proteins were detected only in DENV2-, I-sEVs-, and I-sEVs_AG_-infected mice. As expected, no viral proteins were detected in the negative controls and the DENV2_AG_-infected mice, confirming the absence of replication.

The combined results from RT-qPCR and Immunoblotting analyses suggest that I-sEVs_AG_, which contains inactivated free viral particles, can still promote infection in a neonatal CD-1 mouse model.

### Adult female and male AG129 mice are susceptible to infection mediated by I-sEVs

**Figure 5.**
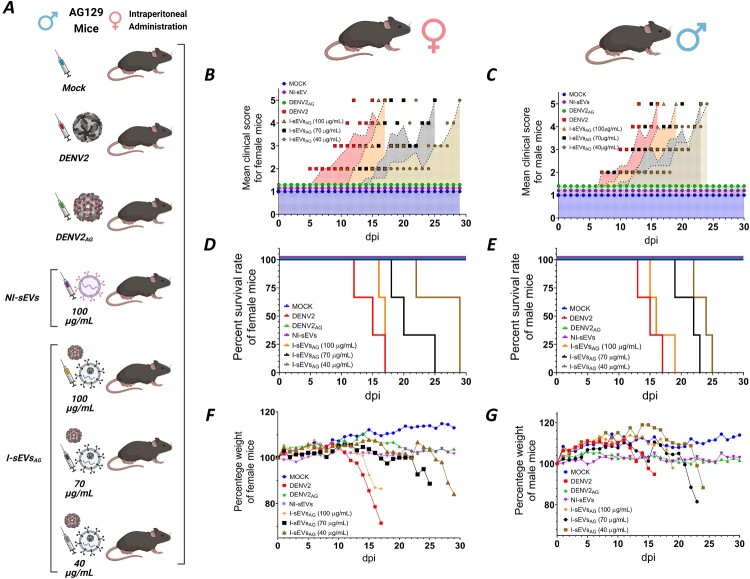
*In vivo,* viral infection mediated by I-sEVs_AG_ in adult AG129 mice (A) Schematic representation of intraperitoneal inoculations performed in adult AG129 mice. Mice were grouped by sex into seven experimental categories: (1) Mock-treated, (2) DENV2-infected (2 × 10⁷ PFU/mL), (3) DENV2_AG_-infected (2 × 10⁷ PFU/mL), (4) 100 µg/mL NI-sEVs, (5) 100 µg/mL I-sEVs_AG_, (6) 70 µg/mL I-sEVs_AG_, and (7) 40 µg/mL I-sEVs_AG_. Diagrams were created using Biorender.com. (B, C) Clinical scores of AG129 mice demonstrate delayed symptom onset in (B) female and (C) male mice treated with all concentrations of I-sEVs_AG_ compared to DENV2 – infected controls. Mock-treated, NI-sEVs, and DENV2_AG_ groups remained asymptomatic throughout the experiment. Each aligned symbol represents the clinical score of an individual mouse according to its treatment group. Data are now represented as median ± IQR or with individual animal data points, as appropriate for ordinal data. For visualization, the geometric mean of each group is connected by a colored dotted line. (D, E) Kaplan-Meier survival curves show the percentage survival of (D) female and (E) male AG129 mice. I-sEVs_AG_-infected mice exhibited increased survival time compared to DENV2-infected mice. DENV2 and all I-sEVs_AG_ concentrations were lethal, while Mock-treated, NI-sEVs-, and DENV2_AG_-infected mice survived the entire experiment. (F, G) The average body weight percentage of (F) female and (G) male AG129 mice reveals a transient weight reduction in all I-sEVs_AG_-infected groups. Mock-treated, NI-sEVs-, and DENV2_AG_-infected mice did not exhibit significant weight loss during the experiment. Statistical comparisons were performed using the Gehan-Breslow Wilcoxon and Log-rank (Mantel-Cox) tests. *P*-values are provided in Table 2. The results shown in the complete figure represent three independent experiments.

Finally, to evaluate the susceptibility of a larger animal model to infection with I-sEVs_AG_, adult AG129 mice were used (Figure 5). Mice were grouped by sex into seven categories (Figure 5A). Mouse weight and signs of disease were monitored daily until the day of euthanasia. The disease progression of female (Figure 5B) and male (Figure 5C) mice was assessed using the clinical scoring system previously described, which has been applied in various studies (Table 1). Additional parameters, such as survival time (Figure 5D–E) and weight loss (Figure 5F–G), were also analyzed.

We observed that inoculation of Mock-treated, NI-sEVs-infected, and DENV2AG-infected mice did not induce any signs of disease in either female (Figure 5B) or male mice (Figure 5C); thus, they were classified as “1” according to the clinical signs table. Clinical signs of DENV2 infection in our positive control group appeared at 6 dpi in female mice and 7 dpi in male mice. Clinical signs, classified as “5” in the clinical signs table, occurred at 15 dpi in female mice and 14 dpi in male mice. In mice infected with 100 µg/mL of I-sEVs_AG,_ clinical signs appeared at 11 dpi in females and 8 dpi in males, respectively. For the 70 µg/mL group, signs appeared at 14 dpi in females and 10 dpi in males. In the 40 µg/mL group, clinical signs appeared at 16 dpi in females and 12 dpi in males. The progression to severe clinical signs was observed in both female and male I-sEVs_AG_-infected mice. Severe signs appeared at 16.6 dpi in females and 17.5 dpi in males for the 100 µg/mL group: 21 dpi in females and 19 dpi in males for the 70 µg/mL group, and 27 dpi in females and 24 dpi in males for the 40 µg/mL group. These results indicate that the I-sEVs_AG_ are infective and produce severe symptoms in female and male AG129 mice (Figure 5B–C). Furthermore, male mice appeared to be more susceptible to infection.

Although the categories’ symptoms have already been described, we observed additional symptoms in our experiments. Therefore, we propose a new clinical morbidity scale for AG129 mice infected with DENV2, as detailed in Table 1. Notably, all infected mice (both female and male) exhibited increased mobility and hyperventilation, classified as “2”, preceding the development of a hunched posture and lethargy. During signs of lethargy, head tilt changes, maintaining the head´s rotation to one side, with closure (total or partial) of one eye (panuveitis) by grey material, classified as “3”. The emergence of more severe symptoms, including alopecia, closed and/or cloudy eyes, and paralysis, was explicitly observed in mice infected with I-sEVs_AG_. When analyzing 3 mice per condition infected with I-sEVs_AG_ (9 mice total), we observed the following symptoms in female mice: alopecia (1/9), closed eyes (7/9), cloudy eyes (1/9), and paralysis (4/9). In male mice, the symptoms included alopecia (2/9), closed eyes (8/9), cloudy eyes (2/9), and paralysis (4/9). In both sexes, the mice most severely affected were those infected with 40 µg/mL I-sEVs_AG_, which presented all the severe symptoms mentioned above. These findings suggest that infection with the lowest concentration of I-sEVs_AG_ induces DENV infection, characterized by new symptoms that affect additional organs, including the skin, eyes, and brain. We also evaluated the survival rate in female (Figure 5D) and male (Figure 5E) mice during I-sEVs_AG_ infection. To determine a significant difference, the survival curves were compared using Kaplan-Meier analysis, which revealed a significant increase in survival in females and males infected with 40 and 70 µg/mL I-sEVs_AG_ compared to the positive control (Table 2). In the 70 µg/mL I-sEVs_AG_ group, the median survival was extended from 15 to 20 dpi in female mice and from 14 to 22 dpi in male mice. Similarly, in the 40 µg/mL I-sEVs_AG_ group, the median survival of female and male mice differs significantly from 15 to 29 and 14–24 dpi, respectively. These results suggest that inoculation with I-sEVs_AG_ can promote infection, with the onset of early and severe disease symptoms, and the lowest concentration (40 µg/mL I-sEVs_AG_) showing the most severe results. We also assessed changes in body weight in female (Figure 5F) and male (Figure 5E) mice during I-sEVs_AG_ infection. Mock-treated, DENV2_AG_-infected, and NI-sEVs-infected female and male mice exhibited no significant changes in body weight throughout the experiments. In female mice, DENV2 infection caused a drastic weight reduction of 28.58%, while I-sEVs_AG_-infected mice showed milder decreases of 13.55% at 100 µg/mL, 11.37% at 70 µg/mL, and 15.92% at 40 µg/mL. All groups initially gained weight in male mice, followed by a subsequent weight decline. Considering their maximum weight peak, DENV2-infected male mice experienced a 16.65% reduction, while I-sEVs_AG_-infected mice showed decreases of 15.89% at 100 µg/mL, 29% at 70 µg/mL, and 30.56% at 40 µg/mL, exhibiting the most pronounced weight change.

**Table 2.** Summary of statistical data for the in vivo assay in adult AG129 female and male mice.

Comparison of groups		# Events (n)	Median Survival (days)	Average survival rate (days)
Females	DENV2 vs. 100 µg/mL I-sEVs_AG_	DENV2 = 3 100 µg/mL I-sEVs_AG_ = 3	DENV2 = 15 100 µg/mL I-sEVs_AG_ = 17	NO Log rank (Mantel-Cox) test *p = 0.3018 NO Gehan-Breslow Wilcoxon test *p = 0.2386
	DENV2 vs. 70 µg/mL I-sEVs_AG_	DENV2 = 3 70 µg/mL I-sEVs_AG_ = 3	DENV2 = 15 70 µg/mL I-sEVs_AG_ = 20	YES Log rank (Mantel-Cox) test *p **=** 0.0246 YES Gehan-Breslow Wilcoxon test *p **=** **0.0339**
	DENV2 vs. 40 µg/mL I-sEVs_AG_	DENV2 = 3 40 µg/mL I-sEVs_AG_ = 3	DENV2 = 15 40 µg/mL I-sEVs_AG_ = 29	YES Log rank (Mantel-Cox) test *p = 0.0246 YES Gehan-Breslow Wilcoxon test *p **=** **0.0339**
Males	DENV2vs. 100 µg/mL I-sEVs_AG_	DENV2 = 3 100 µg/mL I-sEVs_AG_ = 3	DENV2 = 14 100 µg/mL I-sEVs_AG_ = 16	NO Log rank (Mantel-Cox) test *p = 0.4048 NO Gehan-Breslow Wilcoxon test *p = 0.3827
	DENV2 vs. 70 µg/mL I-sEVs_AG_	DENV2 = 3 70 µg/mL I-sEVs_AG_ = 3	DENV2 = 14 70 µg/mL I-sEVs_AG_ = 22	YES Log rank (Mantel-Cox) test *p **=** 0.0246 YES Gehan-Breslow Wilcoxon test *p **=** **0.0339**
	DENV2 vs. 40 µg/mL I-sEVs_AG_	DENV2 = 3 40 µg/mL I-sEVs_AG_ = 3	DENV2 = 14 40 µg/mL I-sEVs_AG_ = 24	YES Log rank (Mantel-Cox) test *p **=** 0.0246 YES Gehan-Breslow Wilcoxon test *p **=** **0.0339**

Words in bold font are highlighted when both statistical tests for *in vivo* assay studies are statistically significant.

**Figure 6.**
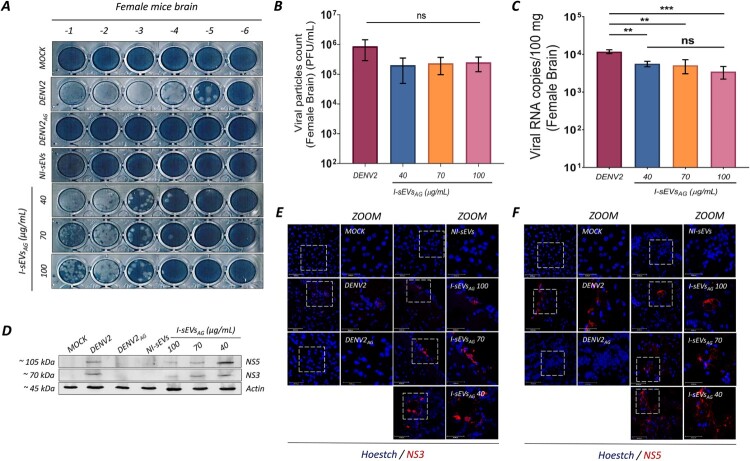
I-sEVs_AG_ enhances viral infection and disease progression in female AG129 mice. (A) Lytic plaque assays demonstrate elevated viral progeny production in brain lysates from I-sEVs_AG_-infected female AG129 mice. Brain lysates from Mock-treated, DENV-, and DENV2_AG_-infected female mice were used as controls. I-sEVs_AG_ did not suppress virion release compared to DENV2_AG_. No virion release was observed in Mock-treated, DENV2_AG_-, or NI-sEVs-infected conditions. (B) Quantification of viral progeny production shows no significant difference between DENV2-infected and I-sEVs_AG_-infected mice across all concentrations tested. Data were represented as mean ± standard error of the mean (SEM). A one-way ANOVA was performed to conduct the statistical comparison, followed by Tukey post hoc tests. Ns = not significant. (C) Real-time PCR quantification reveals increased viral RNA levels in the brains of I-sEVs_AG_-infected female mice at all concentrations. No significant differences in viral RNA levels were observed between I-sEVs_AG_ concentrations. Data were represented as mean ± standard error of the mean (SEM). A one-way ANOVA was performed to conduct the statistical comparison, followed by Tukey post hoc tests. **p = 0.001, ****p = 0.0001. Ns = not significant. (D) Immunoblot analysis confirms the presence of DENV2 NS3 and NS5 proteins in brain lysates from I-sEVs_AG_-infected female mice. Brain lysates from Mock-treated, DENV2-infected, and DENV_AG_-infected female AG129 mice were used as controls. I-sEVs_AG_-infected samples exhibited both viral proteins, while no viral proteins were detected in Mock-treated, DENV2_AG_-, or NI-sEVs-infected conditions. DENV-specific antibodies against NS3 and NS5 were used for detection, with actin as a loading control. (E, F) Immunofluorescence analysis demonstrates viral infection in the brain tissues of I-sEVs_AG_-infected female AG129 mice. Mock-treated, DENV-, and DENV2_AG_-infected female AG129 mice served as controls. All I-sEVs_AG_ concentrations show the presence of (E) NS3 and (F) NS5 viral proteins, with the 40 µg/mL I-sEVs_AG_ concentration exhibiting the highest levels. Viral proteins were detected using DENV-specific antibodies against NS3 and NS5, coupled to anti-rabbit Alexa-555 (red). Nuclei were counterstained with Hoechst (blue): scale bar, 50 µm and 30 nm for ZOOM. The results shown in the complete figure represent two or three independent experiments.

**Figure 7.**
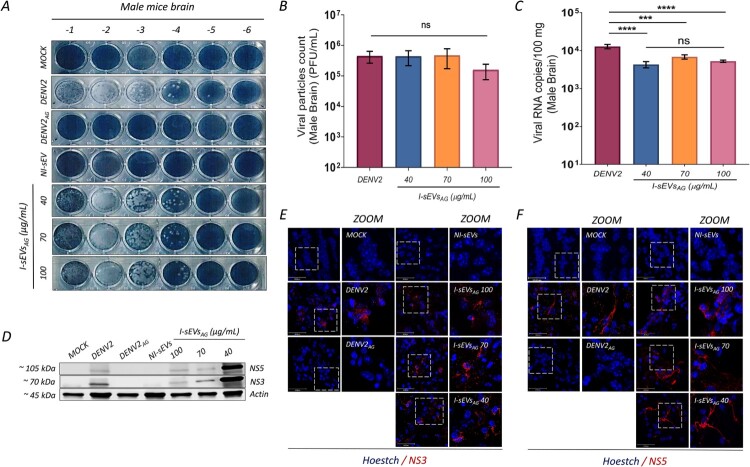
I-sEVs_AG_ enhances viral infection and disease progression in male AG129 mice. (A) Lytic plaque assays demonstrate elevated viral progeny production in brain lysates from I-sEVs_AG_-infected male AG129 mice. Brain lysates from Mock-treated, DENV-, and DENV2_AG_-infected male mice were used as controls. I-sEVs_AG_ did not suppress virion release compared to DENV2_AG_. No virion release was observed in Mock-treated, DENV2_AG_-, or NI-sEVs-infected conditions. (B) Quantification of viral progeny production shows no significant difference between DENV2-infected and I-sEVs_AG_-infected mice across all concentrations tested. Data were represented as mean ± standard error of the mean (SEM). A one-way ANOVA was performed to conduct the statistical comparison, followed by Tukey post hoc tests. Ns = not significant. (C) Real-time PCR quantification reveals increased viral RNA levels in the brains of I-sEVs_AG_-infected male mice at all concentrations. No significant differences in viral RNA levels were observed between I-sEVs_AG_ concentrations. Data were represented as mean ± standard error of the mean (SEM). A one-way ANOVA was performed to conduct the statistical comparison, followed by Tukey post hoc tests. ***p = 0.0001, ****p = <0.0001. Ns = not significant. (D) Immunoblot analysis confirms the presence of DENV2 NS3 and NS5 proteins in brain lysates from I-sEVs_AG_-infected female mice. Brain lysates from Mock-treated, DENV2-infected, and DENV_AG_-infected female AG129 mice were used as controls. I-sEVs_AG_-infected samples exhibited both viral proteins, while no viral proteins were detected in Mock-treated, DENV2_AG_-, or NI-sEVs-infected conditions. DENV-specific antibodies against NS3 and NS5 were used for detection, with actin as a loading control. (E, F) Immunofluorescence analysis demonstrates viral infection in the brain tissues of I-sEVs_AG_-infected female AG129 mice. Mock-treated, DENV-, and DENV2_AG_-infected female AG129 mice served as controls. All I-sEVs_AG_ concentrations show the presence of (E) NS3 and (F) NS5 viral proteins, with the 40 µg/mL I-sEVs_AG_ concentration exhibiting the highest levels. Viral proteins were detected using DENV-specific antibodies against NS3 and NS5, coupled to anti-rabbit Alexa-555 (red). Nuclei were counterstained with Hoechst (blue): scale bar, 50 µm and 30 nm for ZOOM. The results shown in the complete figure represent two or three independent experiments.

Additionally, we investigate viral protein expression, viral load, and viral particle production in the livers and brains of infected mice. Interestingly, no viral protein expression, viral load, or viral particle production was detected in the livers of the mice (data not shown); therefore, subsequent analyses focused exclusively on the brain tissues of infected female (Figure 6) and male (Figure 7) mice. As expected, no viral components were detected in Mock-treated, DENV2_AG_-infected, or NI-sEVs-treated mice (Figure 6A). Viral particle counts demonstrated an increase in the brains of AG129 female mice infected with I-sEVs_AG_ at a concentration of 100 µg/ml (mean 2.50 × 10^4^ PFU/100 mg), 70 µg/ml (mean 2.33 × 10^4^ PFU/100 mg), and 40 µg/ml (mean 2.00 × 10^4^ PFU/100 mg) compared to internal controls. However, these levels were lower, though not statistically significant, than the DENV2-positive control group (mean 8.66 × 10^4^ PFU/100 mg) (Figure 6B). Similarly, in male mice, no viral components were detected in Mock-treated, DENV2_AG_-infected, or NI-sEVs-treated mice (Figure 7A). Viral particle counts increased following infection with I-sEVs_AG_ at concentrations of 100 µg/ml (mean 1.58 × 10^4^ PFU/100 mg), 70 µg/ml (mean 4.73 × 10^4^ PFU/100 mg), and 40 µg/ml (mean 4.41 × 10^4^ PFU/100 mg) compared to internal controls but remained lower without significance relative to the DENV2-positive control group (mean 4.5 × 10^4^ PFU/100 mg) (Figure 7B).

RT-qPCR analysis revealed a significant increase in viral copy numbers in the brains of female AG129 mice infected with I-sEVs_AG_ at a concentration of 100 µg/ml (mean 3.50 × 10^3^ copies of viral RNA/ 100 mg), 70 µg/ml (mean 5.14 × 10^3^ copies of viral RNA/ 100 mg) and 40 µg/ml (mean 5.62 × 10^3^ copies of viral RNA/ 100 mg) compared to internal controls. However, these levels were lower than those observed in the DENV2-positive control group (mean 1.18 × 10^4^ copies of viral RNA/ 100 mg) (Figure 6C). Similarly, results were observed in male AG129 mice infected with I-sEVs_AG_ at 100 µg/ml (mean 5.24 × 10^3^ copies of viral RNA/ 100 mg), 70 µg/ml (mean 6.84 × 10^3^ copies of viral RNA/ 100 mg) and 40 µg/ml (mean 4.30 × 10^3^ copies of viral RNA/ 100 mg) relative to internal controls, compared to the DENV2-positive control group (mean 1.28 × 10^4^ copies of viral RNA/ 100 mg) (Figure 7C). Interestingly, we observed no significant differences in viral copy numbers among the three concentrations of I-sEVs_AG_, in either female or male mice.

Immunoblotting assays detected the presence of NS3 and NS5 viral proteins in both the positive control (DENV2-infected mice) and in all conditions infected with I-sEVs_AG_ across female and male mice. Among the I-sEVs_AG_-infected groups, a significant increase in NS3 and NS5 viral protein levels was observed in the 40 µg/mL condition compared to higher concentrations in female (Figure 6D) and male mice (Figure 7D). Immunofluorescence analysis of frozen brain sections further confirmed these findings. Confocal microscopy revealed that NS3 and NS5 proteins localize to the cytoplasm of infected cells. Interestingly, brain tissues from AG129 mice infected with 40 μg/ml I-sEVs_AG_ exhibited the highest levels of NS3 and NS5 in female (Figure 6E–F) and male mice (Figure 7E–F), suggesting enhanced viral protein expression at this concentration. These findings demonstrate that infection with I-sEVs in female and male mice is lethal, with the onset of severe symptoms marked by the possible accumulation of viral proteins.

This study shows that mosquito cells can generate I-sEVs that contain viral components, and that these sEVs can interact with mammalian cells. At the same time, the content of I-sEVs (in the absence of free viral particles inactivated with AG) induces infection *in vitro* and *in vivo*, characterized by classical DENV2 symptomatology, accompanied by DENV2 genome replication, viral protein expression, and viral particle production in the brain, confirming that I-sEVs can exacerbate the neurological pathological process in a specific immunodeficient model. Notably, new symptoms suggest that I-sEVs may facilitate viral infection in additional tissues, such as the eyes and skin, contributing to DENV2 pathogenesis.

## Discussion

One of the most critical steps in the DENV transmission cycle occurs during the infectious mosquito bite, when saliva containing the virus is co-injected into human skin. Viruses enhance their own survival by modulating arthropod vector-derived salivary factors, which include a mixture of proteins [22] and lipids [23] that alter human cell function, increasing disease severity, and enhancing arbovirus pathogenicity [24–26]. These factors may be secreted directly or packaged into extracellular vesicles (EVs), which are subsequently released into the saliva.

Studies have demonstrated that many viruses exploit these pathways to transport viral components, thereby diversifying their propagation mechanisms and pathological effects [27]. Recent studies have highlighted the ability of *Aedes*-derived sEVs, a well-documented component of mosquito saliva, to facilitate *Orthoflavivirus* transmission, demonstrating their role in *in vitro* [8,9,11,28] and more recently *in vivo* [23] infections. However, their function *in vivo* DENV infection models remains poorly understood. This study proposes that sEVs derived from DENV-infected mosquito cells can propagate DENV2 *in vivo*, resulting in severe DENV-like symptoms.

The generation and secretion of sEVs is a multistep process influenced by pathological conditions, including an altered cellular microenvironment or viral infection [29–31]. Our findings demonstrate that mosquito cells contain MVBs, which are essential for the formation of sEVs at 48 hpi without the formation of cell syncytia (Fig. S1). These MVBs were present in MOCK– and DENV-infected conditions (Figure 1A), with a significant increase in their number following DENV infection, thereby facilitating increased secretion of sEVs.

The increase in MVB is associated with enhanced sEVs production, driven by several cellular mechanisms triggered during DENV infection, including endoplasmic reticulum (ER) stress responses [32], calcium imbalance [33], and protein kinase activity. These responses favor cell survival and enable continued viral transmission to new hosts. Such stress-induced changes can also increase cells’ susceptibility to generate and release sEVs.

Our results show that DENV infection markedly increases both the diameter (Figure 1G) and overall size (Figure 1H) distribution of sEVs released by C6/36 cells, suggesting that infection promotes the incorporation of additional molecular cargo. This may include cellular [10] and viral proteins [8,34], as well as viral components such as subgenomic flavivirus RNA [11]; viral genome [8], and even virus-like particles [9].

Consistent with this, we identified multiple viral components within I-sEVs, including viral RNA (Figure 2B), virus-like particles (Figure 2C), and several viral proteins (Figure 2A), which may play an essential role in sEV-mediated infection in both vertebrate and mammalian cells [35].

Among these viral proteins, NS1, NS3, and NS5 were consistently detected. These proteins are essential for viral replication but may also exert functions in non-infected recipient cells. NS1, a secreted virulence factor [36], together with the replication-associated proteins NS3 and NS5, can modulate cellular pathways and immune responses even in the absence of productive infection [37–39]. While the association of NS1 with sEVs has been previously reported [34], this study provides evidence that NS3 and NS5 are also linked to mosquito-derived I-sEVs (Figure 2A). Their presence within sEVs highlights a potential mechanism by which DENV may alter the physiology of uninfected cells and broaden its pathogenic impact, even across biological barriers such as the blood–brain barrier [40,41]. The detection of viral genomes and proteins in mosquito-derived sEVs suggests that these vesicles act as unconventional carriers of viral replication machinery and immunomodulatory factors, raising the possibility that sEVs may serve as a parallel route of viral dissemination, capable of priming distant tissues even before viral particles arrives. Further studies are needed to determine how these viral components are packaged into sEVs and how they contribute to sEVs-mediated dissemination and host modulation.

The release of different EVs subpopulations during viral infections modulates host responses, which can either promote or inhibit viral activity depending on the context [42,43]. Previous reports have shown that DENV infection reshapes the cargo and function of EVs. Notably, sEVs released from DENV-infected dendritic cells carry distinct RNA (mRNA and miRNA) profiles depending on the strain and clinical severity, suggesting that sEVs contribute to immune modulation during dengue infection [44]. Similarly, sEVs isolated from DENV-infected mosquito cells, selected through surface markers, have been shown to contain viral proteins [34], a complete viral genome [8], and virus-like particles [9] and to infect naïve cells, supporting a role for arthropod-derived EVs in viral dissemination. Our results expand these observations by demonstrating that mosquito-derived sEVs can also deliver viral components into mammalian systems and contribute to disease-associated phenotypes.

Although a specific fraction of sEVs derived from DENV-infected mosquito cells has been shown to possess infective properties [8,9,34], the full pathogenesis role of all sEVs subpopulations remains unclear. Therefore, in this study, we chose to examine all subpopulations of sEVs.

To accurately assess their global effects, viral particles had to be inactivated. Acid glycine (AG) treatment, previously used to block viral entry in mosquito [45,46] and mammalian cells [47,48], by inducing irreversible conformational changes [49,50]. Using a modified version of Hung’s protocol [48], we confirmed that AG efficiently inactivates viral particles, blocking entry, replication (Fig. S3B-C), and progeny production (Fig. S3D). Significantly, AG treatment did not alter I-sEVs morphology (Figure 3A) and preserved their ability to enter Huh-7 cells (Figure 3B), supporting viral replication (Figure 3C) and particle release (Figure 3D).

Methodologies, such as RNase and proteinase K, can confirm that viral infection is derived from the contents of sEVs. We did not employ these approaches because they can disrupt possible factors essential for efficient sEVs uptake. Proteolytic removal of membrane proteins (including tetraspanins, adhesion molecules, and ligand – receptor pairs) markedly reduces binding, uptake efficiency [7,52], and vesicle internalization [40,51]. Likewise, RNase exposure can eliminate surface-associated RNAs that contribute to sEVs – cell interactions and functional cargo transfer [53]. Because our objective was to evaluate the intrinsic ability of sEVs to promote infection in target cells, applying enzymatic treatments that compromise receptor engagement would confound the interpretation of our results. Future studies using receptor-blocking assays, identification of sEVs uptake receptors, and in-depth profiling of sEVs cargo will help elucidate the mechanisms underlying sEVs-mediated infection and dissemination.

To further explore the infectious potential of I-sEVs, we evaluated their effects *in vivo* models. We selected CD-1 neonatal mice as our model system due to their suitability for DENV replication via intracranial (i.c.) inoculation [16] (Figure 4). Our results showed that infection with DENV2, I-sEVs, and I-sEVs_AG_ caused symptoms such as turning over, imbalance, trembling, and claudication. These symptoms align with previous observations in neonatal CD-1 mice infected with neurotropic viruses [54]. Increased viral copy numbers in infected mice confirmed viral replication (Figure 4C). While DENV2_AG_-infected mice exhibited no symptoms, we detected viral genome remnants in their brain, suggesting that AG treatment, which disrupts viral particle structure, is sufficient to prevent viral replication in CD-1 mice (Figure 4D).

We utilized AG129 mice deficient in IFN-α/β and – γ receptors to further investigate infectivity on the 129/Sv genetic background. These widely used mice are a robust model for DENV infection due to their ability to support high levels of DENV replication [55]. Interestingly, we observed significant sex-dependent differences, particularly at lower I-sEV concentrations, with male mice being more affected (Figure 5C, E, G). Although this sex-based variation was not a planned component of our experimental design, we hypothesized that biological responses in mice depend on the concentration of sEVs inoculated [56]. Specifically, lower concentrations of I-sEVs_AG_ result in gradual infection and symptom development, potentially allowing I-sEVs to reach additional organs due to their intrinsic properties. We determined, at least indirectly, that the kinetics of I-sEVs infection are slower than those of DENV2 in *in vitro* assays (Fig. S4), and this is likely to also be the case in *in vivo* assays. However, further studies are needed to fully elucidate this phenomenon.

Our findings indicate that both female and male mice develop disease symptoms associated with severe cases in humans and mice, including neurological, muscular, alopecia, and visual damage, at all concentrations of I-sEVs_AG_, with the effects more pronounced across the lowest concentration (40 μg/mL). This suggests that I-sEVs can go unnoticed by the host, gradually promoting viral infection to other target organs. However, because severe neurological symptoms are exceedingly rare in human dengue infection [57], we emphasize that these phenotypes reflect the unique susceptibility of the AG129 model rather than typical human dengue pathology. Studies using AG129 mice confirm their value as a model for investigating and understanding the neurological manifestations of severe disease, as both adapted and non-adapted neurovirulent DENVs can affect the brain in these mice [58]. Using CD-1 mouse-adapted DENV2, we generated a virus capable of systemic inoculation in AG129 mice, resulting in neurovirulence after passage through C6/36 mosquito cells. Our results demonstrate brain damage caused by I-sEVs_AG_, which promotes viral replication (Figure 6–7C), virion production (Figure 6–7A-B), and the presence of viral proteins in the brains of AG129 mice (Figure 6–7D–F). We observed additional damage to the lower extremities in experiments in which mouse-adapted DENV2 could cause paralysis in AG129 mice [59]. Further investigations into different viral sublethal doses may avoid systemic damage while leading to paralysis [60], underscoring the importance of examining lower sEVs doses to elucidate their effects on the DENV pathogenesis. In the AG129 mouse model of antibody-dependent enhancement (ADE) of DENV infection, ocular damage is a feature of severe disease. Changes in ocular vascularity, inflammation, and the presence of viral RNA in the retina have all been documented [61]. Unfortunately, the AG129 model has limitations, including profound immunodeficiency, exaggerated viral susceptibility, and poor representation of hemorrhagic dengue phenotypes. These constraints must be considered when extrapolating findings to human disease.

Recently, the role of sEVs in *in vivo* experiments has gained increasing attention [23,56], yet studies involving the direct administration of purified mosquito-derived sEVs remain limited. Our results demonstrate that these I-sEVs can enhance viral infection and induce severe disease-related symptoms by reaching immune-privileged sites. These findings raise the possibility that sEVs may contribute to mosquito-to-human transmission by transporting viral components independently of classical virions, an idea that warrants further dedicated investigation.

### Limitations of the study

In our in vivo experiments, we used intraperitoneal inoculation in AG129 mice, a highly permissive but severely immunodeficient model. Because these mice lack type I and II interferon signaling, they develop neurological manifestations that are exceedingly rare in human dengue. Thus, the severe CNS pathology observed in this study should be interpreted as model-specific rather than a direct representation of severe dengue in humans. Moreover, the physiological dose, composition, and natural delivery of mosquito-derived sEVs remain unknown. The number of sEVs introduced during a mosquito bite – as well as their ratio to infectious virions – has not been quantified. Therefore, the concentrations used in this study may not accurately reflect natural exposure, and future work using DENV-infected mosquitoes will be necessary to determine the relevance of sEV-mediated transmission under physiological conditions.

## Resource availability

### Lead contact

Further information and requests for resources and reagents should be directed to and will be fulfilled by the lead contact, Rosa María Del Ángel (rmangel@cinvestav.mx).

## Materials availability

This study did not generate new unique reagents.

## Data  and code availability

- The lead contact will share all the original data reported in this paper upon request.
- This paper does not report the original code.
- Any additional information required to reanalyze the data reported in this paper is available from the lead contact upon request.

## Supplementary Material

Figure S4.tif

Figure S1.tif

Figure S2.tif

EMI_Supplemental_Information_with_author_details-clean.docx

Figure S3.tif
