# Supplementary figures and images for: Small extracellular vesicles from DENV2-infected C6/36 cells show viral infection in vitro and in vivo
Source: Emerg Microbes Infect. 2026 Jan 27;15(1):2608403. doi: 10.1080/22221751.2025.2608403 (PMC12849808; doi:10.1080/22221751.2025.2608403)

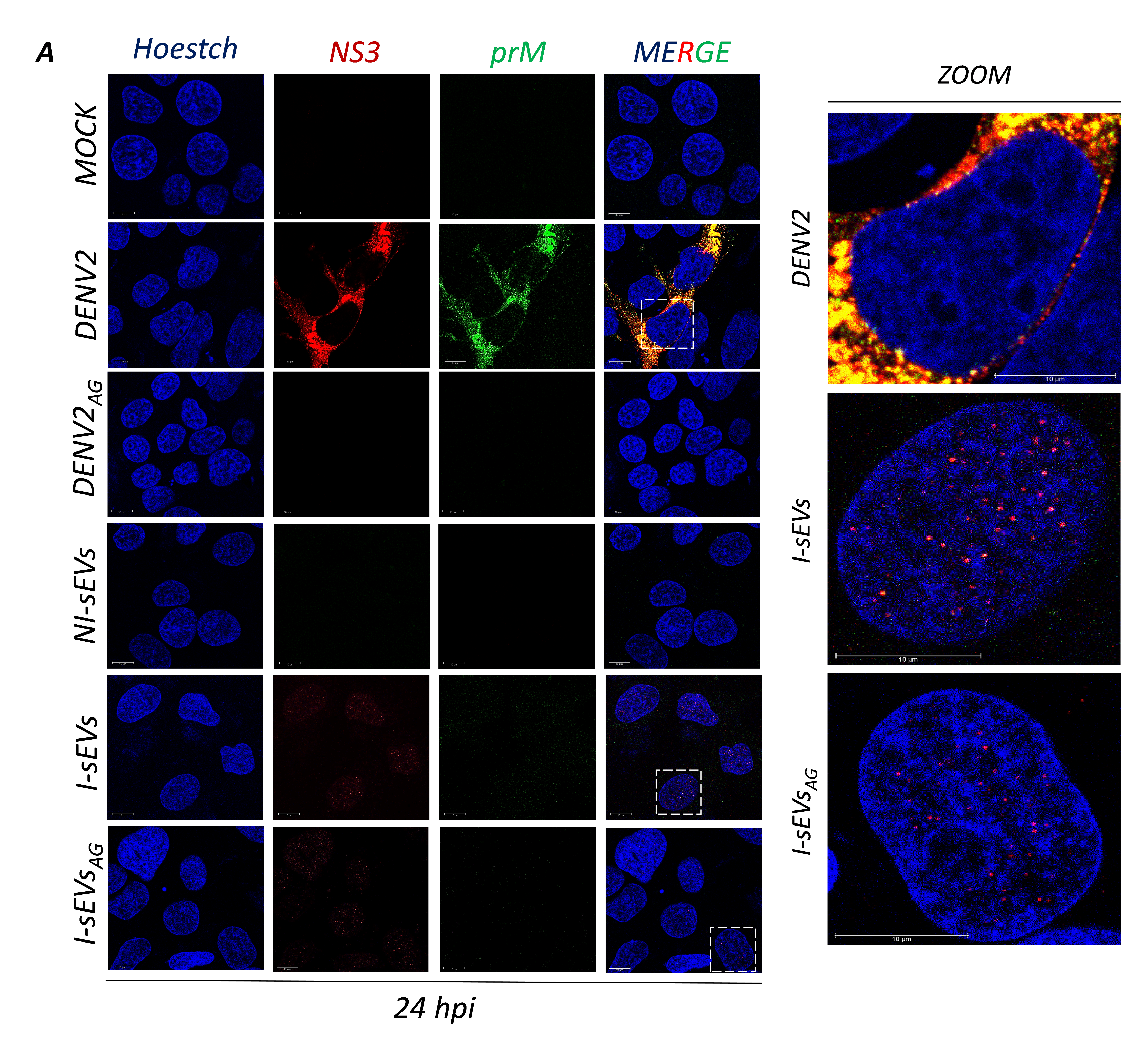

Supplement: Figure S4.tif [file TEMI_A_2608403_SM4359.tif]

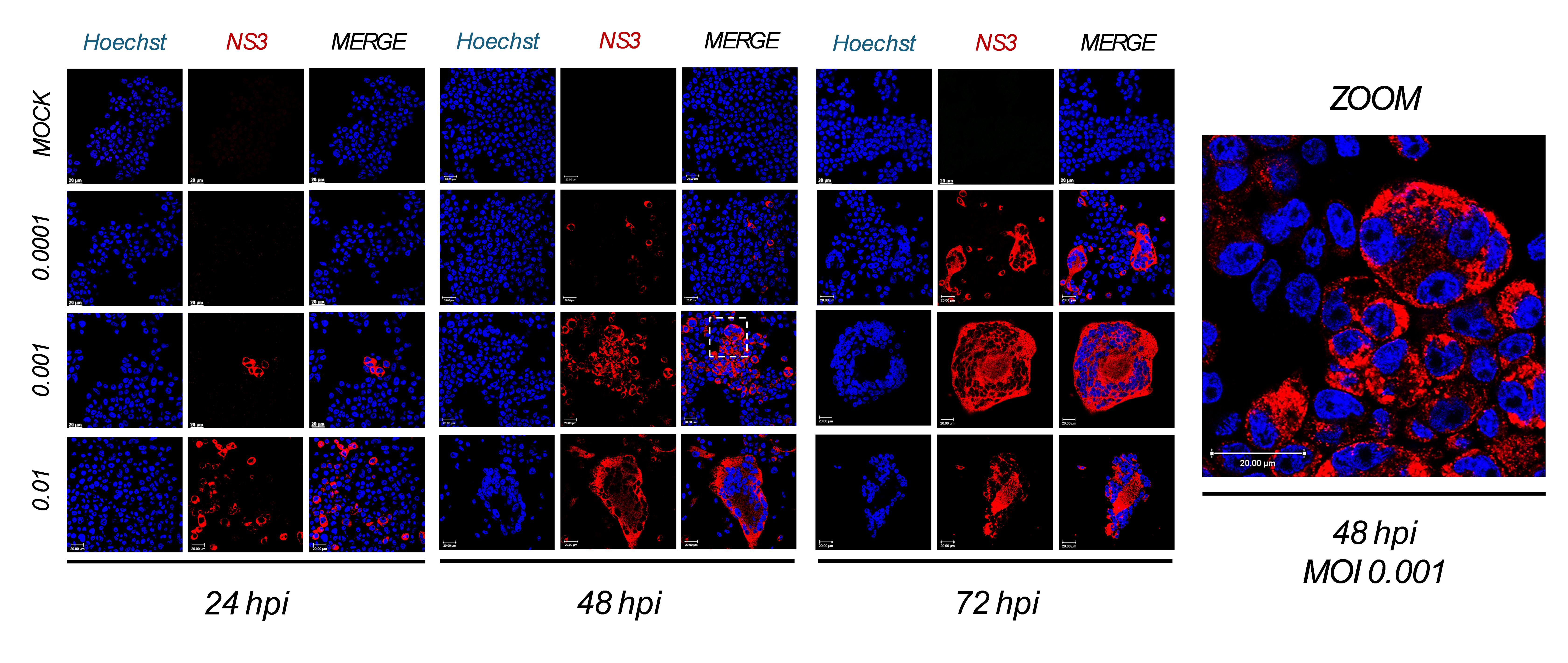

Supplement: Figure S1.tif [file TEMI_A_2608403_SM4358.tif]

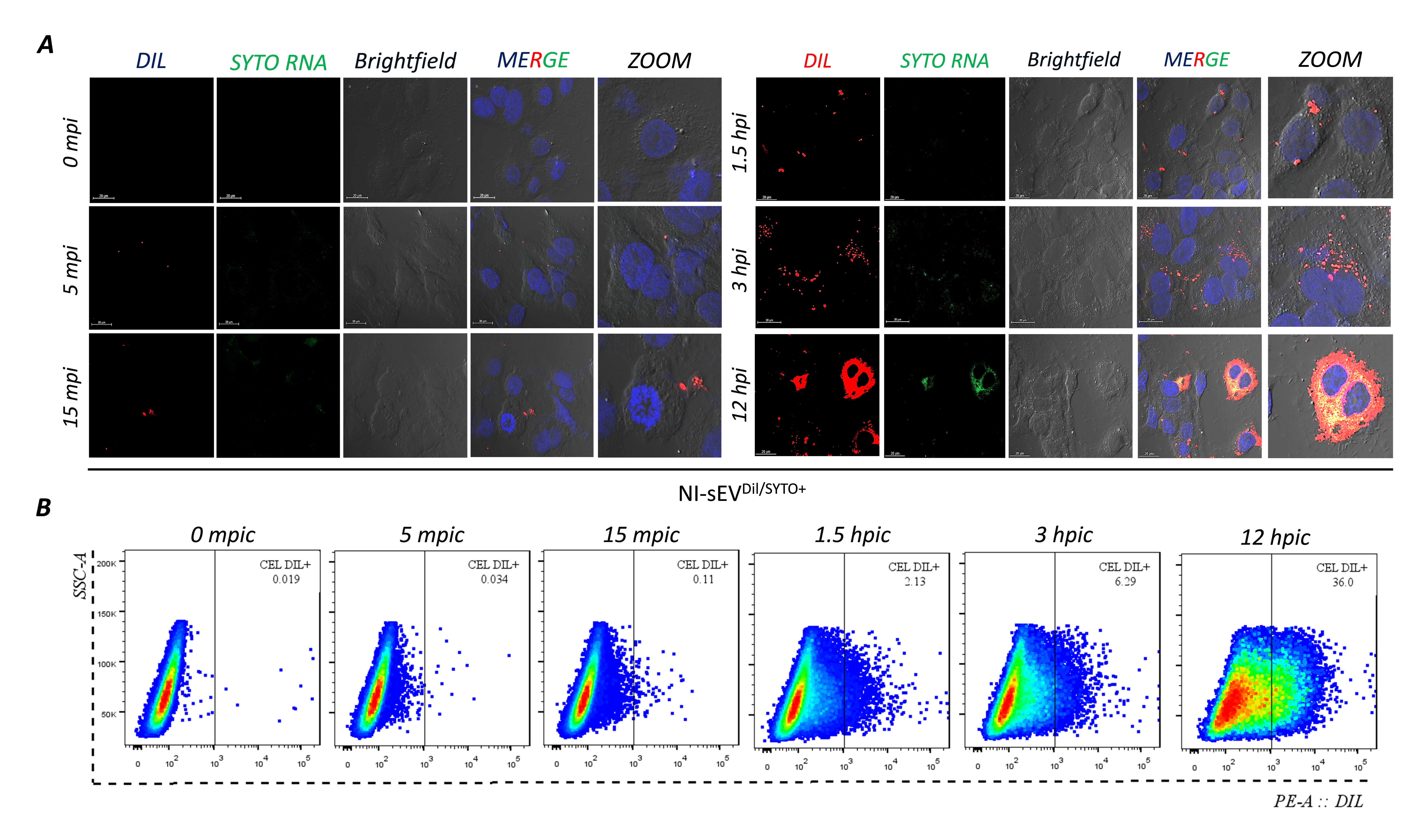

Supplement: Figure S2.tif [file TEMI_A_2608403_SM4357.tif]

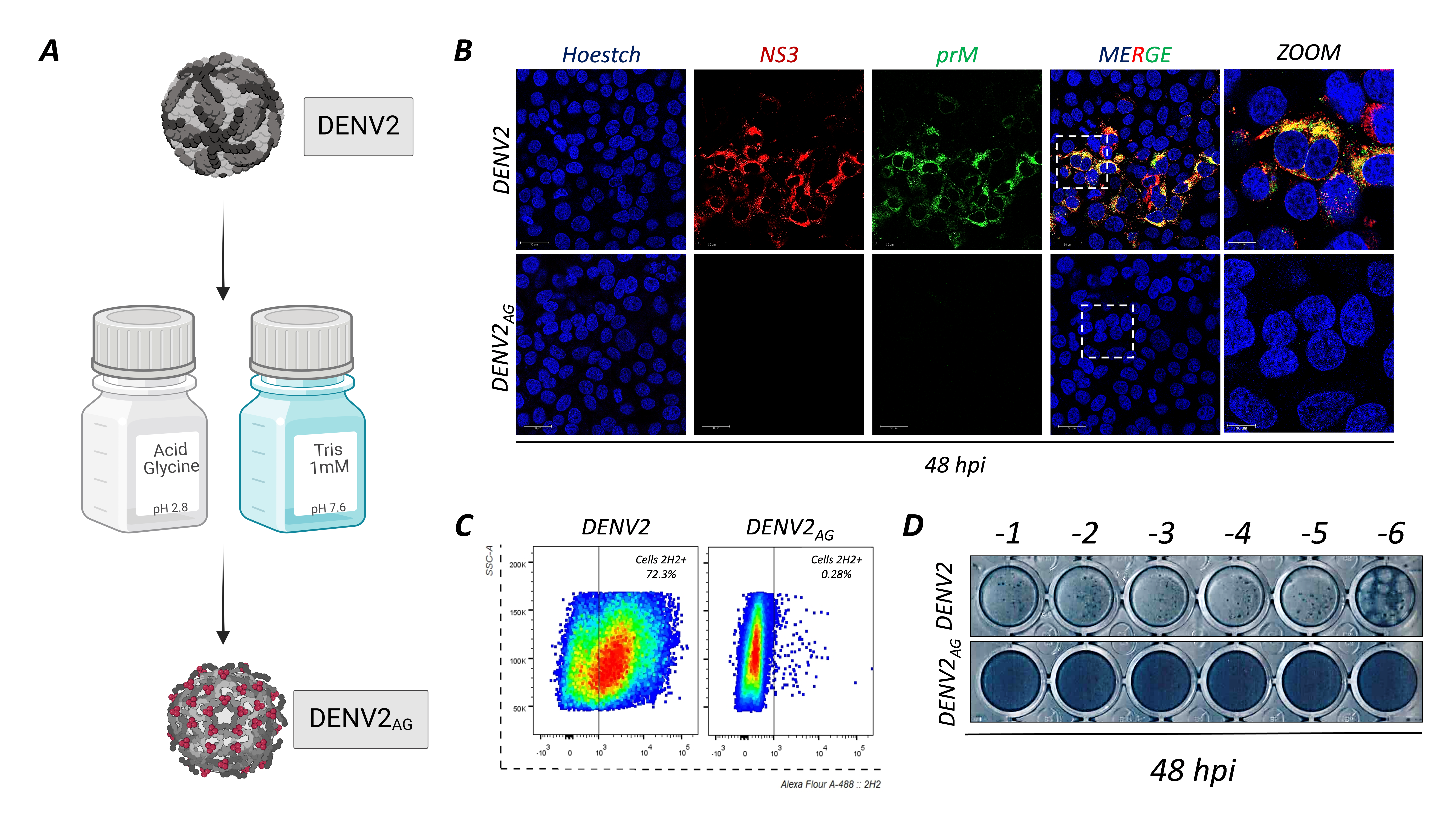

Supplement: Figure S3.tif [file TEMI_A_2608403_SM4355.tif]
