# Supplementary material for: Small extracellular vesicles from DENV2-infected C6/36 cells show viral infection in vitro and in vivo
Source: Emerg Microbes Infect. 2026 Jan 27;15(1):2608403. doi: 10.1080/22221751.2025.2608403 (PMC12849808; doi:10.1080/22221751.2025.2608403)
Supplement: EMI_Supplemental_Information_with_author_details-clean.docx [file TEMI_A_2608403_SM4356.docx]

# **SUPPLEMENTAL INFORMATION**

**Document S1 contains Figures S1–S2 and Table S1**.

**Small Extracellular Vesicles from DENV2-Infected C6/36 Cells Show Viral Infectivity *In Vitro* and *In Vivo***

Carlos D. Cordero-Rivera^1,8^, Magda L. Benítez-Vega^1^, Selvin N. Palacios-Rápalo^2^, José De Jesús Bravo-Silva^1^, Ricardo Jiménez-Camacho^1^, Jonathan Hernández-Castillo^1^, Marcos Pérez-García^1^, Carlos N. Farfan-Morales^1,3^, Luis A. De Jesús-González^4^, José M. Reyes-Ruiz^5^, Juan F. Osuna-Ramos^6^, Fernando Medina-Ramirez^1^, Daniel Talamás-Lara^7^, Raymundo Cruz-Pérez^1^. Arturo Reyes-Sandoval^8^* & Rosa M. Del Angel^1^*

Arturo Reyes-Sandoval^8^* & Rosa M. del Angel^1^*

Email: [arturoreyess@ipn.mx](mailto:arturoreyess@ipn.mx) & [rmangel@cinvestav.mx](mailto:rmangel@cinvestav.mx)

**This section includes:**

Figures S1 to S4

Tables S1

Supporting Information (SI)

- Supporting Figures S1-S4 and legends (Pages 2-5)
- Supporting Table 1 and legend (Pages 6-8)

**Figure supplementary 1**

**
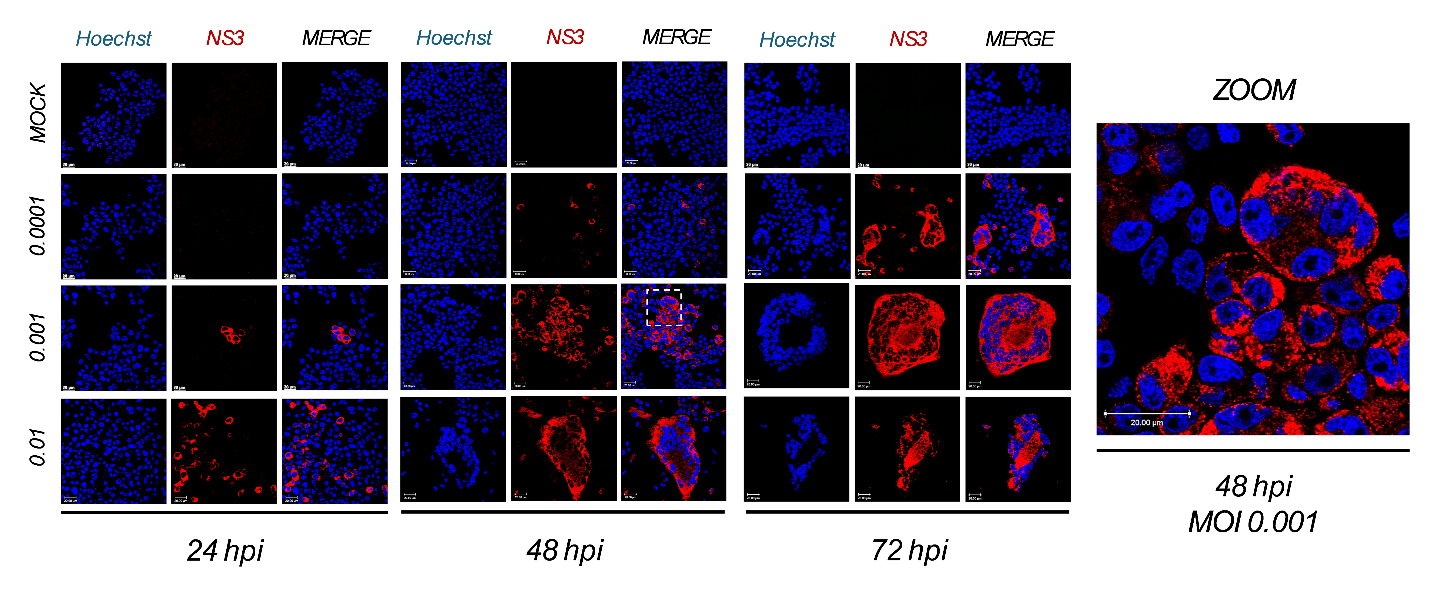
**

Figure S1. C6/36 mosquito cells infected with an MOI of 0.001 for 48 hpi show infection without the formation of cellular syncytia.

Related to Figure 1

Immunofluorescence analysis demonstrates increased infection at 48 hpi using a MOI of 0.001 in mosquito cells, reaching the most significant infection without the formation of cell syncytia. Mock-treated cells served as controls. All conditions (except 24 hpi MOI 0.0001) show the presence of NS3 viral protein. Viral proteins were detected using DENV-specific antibodies against NS3, coupled to anti-rabbit Alexa-555 (red). Nuclei were counterstained with Hoechst (blue). The last column displays a close-up view of individual cells for the selected condition, with a scale bar of 50 µm and 20 nm for ZOOM.

**Figure supplementary 2**


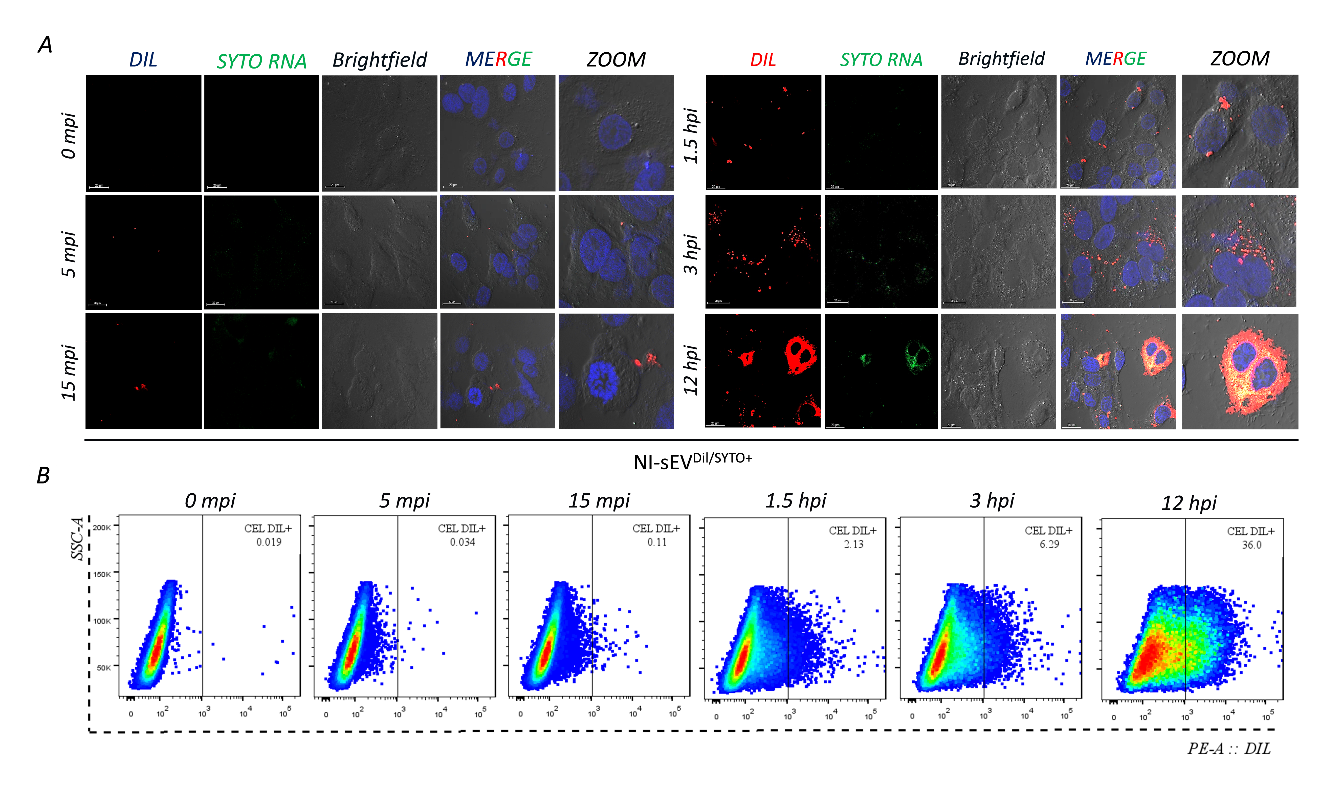


Figure S2. NI-sEVs demonstrate cellular uptake and interaction in Huh-7 cells.

Related to Figure 3

(A) Immunofluorescence analysis demonstrates increased interaction and uptake of NI-sEVs by Huh-7 cells over time in interaction kinetics assays. Freshly isolated NI-sEVs, derived from Mock-treated mosquito cells, were labeled with DiL and SYTO® RNASelect (SYTO RNA) (NI-sEVs^DiL/SYTO+^). Results show increased DiL and SYTO RNA labeling in Huh-7 cells, indicating enhanced interaction and internalization. The last column displays a close-up view of individual cells for each condition. Scale bar, 30 mm. (B) Flow cytometry analysis confirms increased DiL labeling in Huh-7 cells treated with NI-sEVs^DiL/SYTO+^, further supporting the interaction and uptake of NI-sEVs by Huh-7 cells.

**Figure supplementary 3**

**
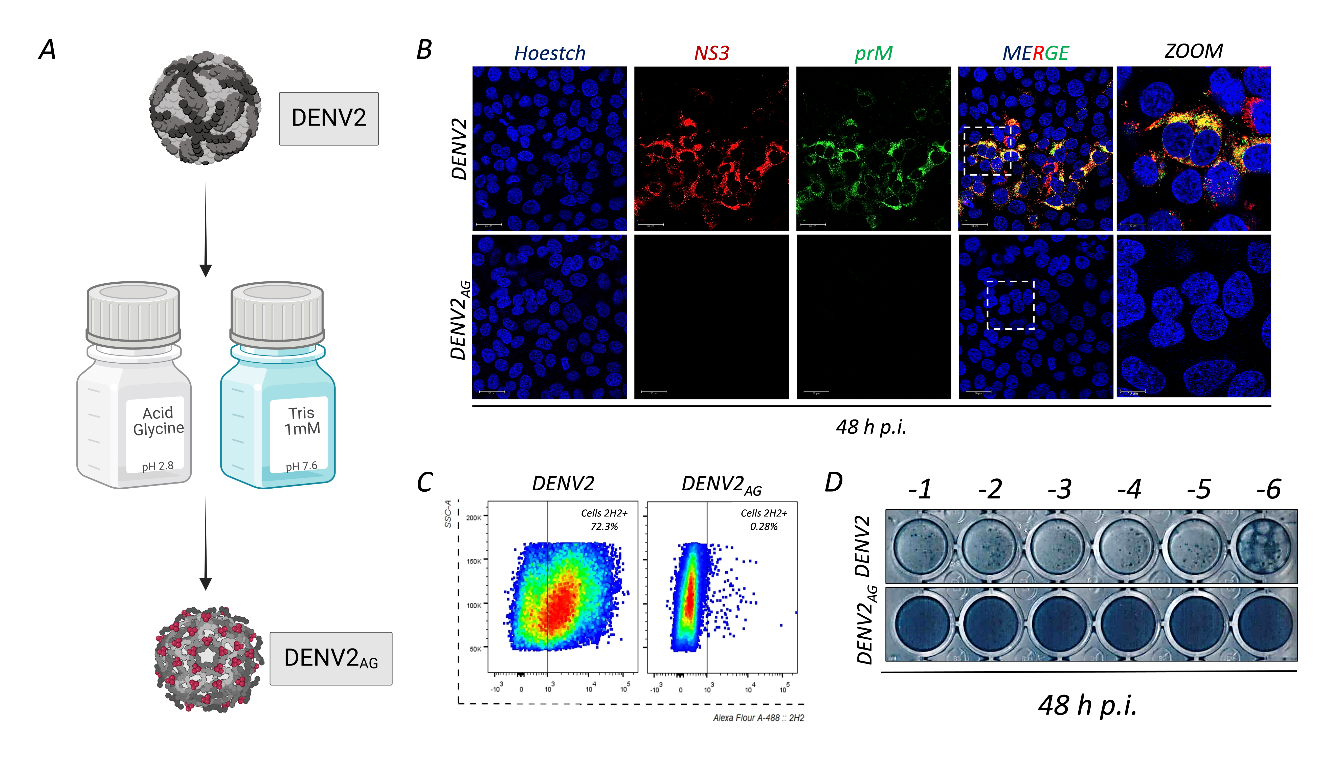
**

Figure S3. Acid glycine (AG) inhibits the entry and replication of dengue viral particles in Huh-7 mammalian cells.

Related to Figure 3

(A) Schematic representation of viral particle inactivation by acid glycine wash. The pH change induces structural alterations on the virion surface, preventing viral entry and replication. (B) Immunofluorescence analysis demonstrates complete inhibition of DENV2_AG_-infection in Huh-7 cells. Huh-7 cells were infected with DENV2 and DENV2_AG_ (MOI 0.001, 48 hpi). No viral proteins were detected in DENV2_AG_-infected cells compared to DENV2-infected cells. Viral proteins were detected using DENV-specific antibodies against NS3 coupled to anti-rabbit Alexa-555 (red) and prM/E coupled to anti-mouse Alexa-488 (green). Nuclei were counterstained with Hoechst (blue). The last column displays a close-up view of individual cells for each condition. Scale bar, 30 mm. (C) Flow cytometry analysis confirms the absence of infected Huh-7 cells following DENV2_AG_ infection. (D) Lytic plaque assays show no virion release in the supernatant of Huh-7 cells infected with DENV2_AG_

**Figure supplementary 4**

**
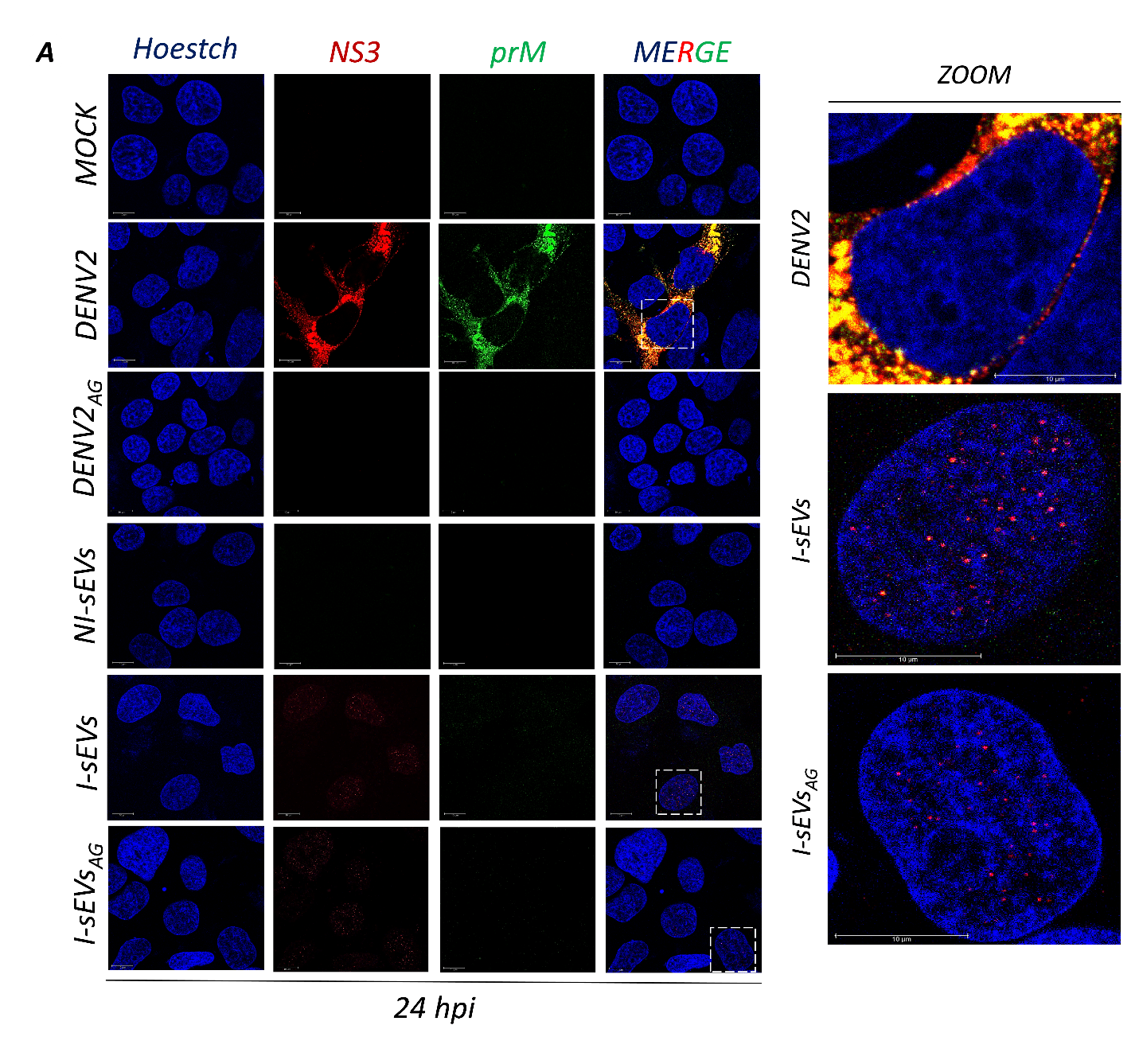
**

Figure S4. The kinetics of infection mediated by I-sEVs_AG_ are slower than those of DENV.

Related to Figure 3 and 5

(A) Immunofluorescence analysis demonstrates a delayed onset of infection in Huh-7 cells treated with 30 µg/mL mediated by I-sEVs_AG_ at 24 hpi. Huh-7 cells treated with mock, infected with DENV, and DENV2_AG_ served as controls. I-sEVs_AG_ shows NS3 protein staining within the nucleus, characteristic of the early stages of cellular infection, while DENV2 already shows staining in the cytoplasm. Viral proteins were detected using DENV-specific antibodies directed against NS3 conjugated to Alexa-555 anti-rabbit (red) and prM/E conjugated to Alexa-488 anti-mouse (green). Nuclei were counterstained with Hoechst (blue). The zoom box displays an enlarged view of individual cells for each condition, accompanied by a 10 µm scale bar.

**TABLES AND TEXT BOXES**

## **Table supplementary 1**

## **Table S1. Reagents used during the preparation of this article.**

| REAGENT OR RESOURCE | SOURCE | IDENTIFIER |  |
| --- | --- | --- | --- |
| Antibodies | | | |
| Mouse anti-CD9 | Santa Cruz Biotechnology | Cat. sc-59140; RRID:AB_1120766 |  |
| Mouse anti-CD63 | Santa Cruz Biotechnology | Cat. sc-365604; RRID:AB_10847220 |  |
| Mouse anti-CD81 | Santa Cruz Biotechnology | Cat. sc-166029; RRID:AB_2275892 |  |
| Mouse anti-annexin V | Santa Cruz Biotechnology | Cat. sc-74438; RRID:AB_1118989 |  |
| Mouse anti-beta actin | Santa Cruz Biotechnology | Cat. sc-81178; RRID:AB_2223230 |  |
| Rabit anti-Capsid | GeneTex | Cat. GTX103343; RRID:AB_1240697 |  |
| Mouse anti-PrM/E | ATCC | Cat. HB-114; RRID:CVCL_J891 |  |
| Rabbit anti-NS1 | Laboratory made | N/A |  |
| Rabbit anti-NS3 | GeneTex | Cat. GTX124252; RRID:AB_11171668 |  |
| Rabbit anti-NS5 | GeneTex | Cat. GTX124253; RRID:AB_11169932 |  |
| Goat anti-mouse Alexa Fluor 488 | Invitrogen | Cat. A21202; RRID: AB_141607 |  |
| Goat anti-rabbit Alexa Fluor 555 | Invitrogen | Cat. A21428; RRID: AB_2535849 |  |
| Bacterial and virus strains | | | |
| DENV-2 New Guinea C Strain | Kindly donated by Instituto de Diagnóstico y Referencia Epidemiológicos Dr. Manuel Martínez Báez (InDRE), Mexico. | N/A |  |
| Chemicals, peptides, and recombinant proteins | | | |
| DMEM | Gibco | Cat. 12491-015 |  |
| MEM | Gibco | Cat. 41500-018 |  |
| 2x Earle´s minimum essential (MEM) | Gibco | Cat. 11935-046 |  |
| HANK’s balanced salt solution | Gibco | Cat. 24020-117 |  |
| Trizol reagent | Invitrogen | Cat. 15596026 |  |
| Super Signal^TM^ West Femto Chemiluminescent Substrate | Thermo Scientific | Cat. 34095 |  |
| SYBR^TM^ Safe DNA Gel Satin | Invitrogen | Cat. S33102 |  |
| Dil stain (1,1'-dioctadecyl-3,3,3,3',3'-tetramethylindocarbocyanine perchlorate ('Dil'; DiIC18(3))) | Invitrogen | Cat. D282 |  |
| SYTO™ RNASelect^TM^ Green Fluorescent Nucleic Acid Stains | Invitrogen | Cat. S32703 |  |
| Hoechst dye | Santa Cruz Biotechnology | Cat. sc-394039 |  |
| Tissue Freezing Medium | Leica | Cat. 14020108926 |  |
| Carboxymethylcellulose sodium salt | Sigma-Aldrich | Cat. C4888 |  |
| Naftol Blue Black (NBB) | Sigma-Aldrich | Cat. N3393 |  |
| Critical commercial assays | | | |
| Total Exosome Isolation Reagent (from culture media) | Invitrogen | Cat. 4478359 |  |
| Pierce BCA protein assay kit | Thermo Scientific | Cat. 23225 |  |
| ImProm-II™ Reverse Transcriptase protocol | Promega | Cat. A3803 |  |
| Recombinant polymerase | Thermo Scientific | Cat. 10342046 |  |
| Experimental models: Cell lines | | | |
| *Aedes albopictus* larvae clone (C6/36 HT) | Kindly donate by Instituto de Medicina Tropical ‘‘Pedro Kourı´’’, Cuba. | N/A |  |
| Human hepatocellular carcinoma (Huh-7) | Kindly donated by Dr. Rivas from Universidad Autonoma de Nuevo Leon, Mexico. | N/A |  |
| Baby hamster kidney-21 (BHK-21) | Kindly donated by Instituto de Medicina Tropical ‘‘Pedro Kourı´’’, Cuba. | N/A |  |
| Experimental models: Organisms/strains | | | |
| CD-1 mice: ICR-CD1, Strain Code: 022. | Charles River Laboratories | N/A |  |
| AG129 mice: strain 129/Sv mice doubly deficient in IFN-α/β and -γ receptors | Marshall Bioresources. | N/A |  |
| Oligonucleotides | | Amplified region | |
| Primer of fragment 1 of the DENV2 genome forward:  5´-AGTTGTTAGTCTACGTGGACCGACA-3´ | Vora et al.^8^ | UTR-5´  prM  C |  |
| Primer of fragment 1 of the DENV2 genome reverse:  5´-TGTCAACCCAGCTTCCTCCTG-3´ | Vora et al.^8^ |  |  |
| Primer of fragment 2 of the DENV2 genome forward:  5´-agccaaacaacctgccactc-3´ | Vora et al.^8^ | E |  |
| Primer of fragment 2 of the DENV2 genome reverse:  5´-GTCCCGGCTCTACTCCTATGATGA-3´ | Vora et al.^8^ |  |  |
| Primer of fragment 3 of the DENV2 genome forward:  5´-GTCAACCCAATCGTAACAGAAAAAGA-3´ | Vora et al.^8^ | E  NS1 |  |
| Primer of fragment 3 of the DENV2 genome reverse:  5´-CTCCAGAGGGTGTGTGACTTTG-3´ | Vora et al.^8^ |  |  |
| Primer of fragment 4 of the DENV2 genome forward:  5´-CGATATGGGTTATTGGATAGAAAGTG-3´ | Vora et al.^8^ | NS1  NS2A |  |
| Primer of fragment 4 of the DENV2 genome reverse:  5´-GTTGGTTCTTGAAAGGGTTGTTAGA-3´ | Vora et al.^8^ |  |  |
| Primer of fragment 5 of the DENV2 genome forward:  5´-CCTCACAGCAGAAAGCGGA-3´ | Vora et al.^8^ | NS2A  NS2B  NS3 |  |
| Primer of fragment 5 of the DENV2 genome reverse:  5´-GACTATGGCCGGAAGGTATCTCT-3´ | Vora et al.^8^ |  |  |
| Primer of fragment 6 of the DENV2 genome forward:  5´-CAATCCAGAGATCGAAGATGACA-3´ | Vora et al.^8^ | NS3 |  |
| Primer of fragment 6 of the DENV2 genome reverse:  5´-GCCTTCAGCTGCCACTCTGT-3´ | Vora et al.^8^ |  |  |
| Primer of fragment 7 of the DENV2 genome forward:  5´-CAGAGCGTGAAAAGGTGGATG-3´ | Vora et al.^8^ | NS3  NS4A  NS4B |  |
| Primer of fragment 7 of the DENV2 genome reverse:  5´- GAGCTGCTGTGAGAGTTATGGGGT -3´ | Vora et al.^8^ |  |  |
| Primer of fragment 8 of the DENV2 genome forward:  5´- GTTAATGGGTCTTGGGAAAGGA -3´ | Vora et al.^8^ | NS4B  NS5 |  |
| Primer of fragment 8 of the DENV2 genome reverse:  5´-GGCTCCTCCATATTTCCTTTGTAGT-3´ | Vora et al.^8^ |  |  |
| Primer of fragment 9 of the DENV2 genome forward:  5´-CCCAATTTTGCATAAAGGTTCTCA-3´ | Vora et al.^8^ | NS5 |  |
| Primer of fragment 9 of the DENV2 genome reverse:  5´-CTTCTAGTGTGATTCTTGTGTCCCA-3´ | Vora et al.^8^ |  |  |
| Primer of fragment 10 of the DENV2 genome forward:  5´-CTTGAGTGGAGTGGAAGGAGAAG-3´ | Vora et al.^8^ | NS5 |  |
| Primer of fragment 10 of the DENV2 genome reverse:  5´-CCCAATCAATGAGCCGCA-3´ | Vora et al.^8^ |  |  |
| Primer of fragment 11 of the DENV2 genome forward:  5´-GGAACAGGGTGTGGATTCAAGA-3´ | Vora et al.^8^ | UTR-3´ |  |
| Primer of fragment 11 of the DENV2 genome reverse:  5´-AGAACCTGTTGATTCAACAGCACCA-3´ | Vora et al.^8^ |  |  |
| Primer Huh-7 GAPDH gene forward:  5´-CTCTGATTTGGTCGTATTGG-3´ | NCBI (NM_001256799.3) | N/A |  |
| Primer Huh-7 GAPDH gene reverse:  5´-GTAAACCATGTAGTTGAGGTC-3´ | NCBI (NM_001256799.3) | N/A |  |
| Primer DENV Capsid gene Forward:  5´-CAATATGCTGAAACGCGAGA-3´ | Prada-Arismendy et al.^60^ | N/A |  |
| Primer DENV Capsid gene Reverse:  5´-TGCTGTTGGTGGGATTGTTA-3´ | Prada-Arismendy et al.^60^ | N/A |  |
| Software and algorithms | | | |
| GraphPad Prism 8.0 | GraphPad Software, San Diego California USA | <https://www.graphpad.com> |  |
| FlowJo v. 10 software | BD Life Sciences | <https://www.flowjo.com/solutions/flowjo> |  |
| Leica Application Suite X Core v3.3.0 | Leica | https://www.leica-microsystems.com/ products/microscope-software/details/ product/leica-las-x-ls/ |  |
| EcoStudy v.5.0 | Illumina | https://support.illumina.com/downloads/eco_real_time_pcr_software_v5-0.html |  |
